# The genetic architecture of genome‐wide recombination rate variation in allopolyploid wheat revealed by nested association mapping

**DOI:** 10.1111/tpj.14009

**Published:** 2018-07-19

**Authors:** Katherine W. Jordan, Shichen Wang, Fei He, Shiaoman Chao, Yanni Lun, Etienne Paux, Pierre Sourdille, Jamie Sherman, Alina Akhunova, Nancy K. Blake, Michael O. Pumphrey, Karl Glover, Jorge Dubcovsky, Luther Talbert, Eduard D. Akhunov

**Affiliations:** ^1^ Department of Plant Pathology Kansas State University Manhattan KS USA; ^2^ USDA‐ARS Cereal Crops Research Unit 1605 Albrecht Blvd N Fargo ND USA; ^3^ INRA GDEC Auvergne‐Rhône‐Alpes Clermont‐Ferrand France; ^4^ Montana State University Bozeman MT USA; ^5^ Integrated Genomics Facility Kansas State University Manhattan KS USA; ^6^ Department of Crop and Soil Sciences Washington State University Pullman WA USA; ^7^ Department of Agronomy, Horticulture and Plant Science South Dakota State University Brookings SD USA; ^8^ Department of Plant Sciences University of California Davis, Davis CA USA; ^9^ Howard Hughes Medical Institute Chevy Chase MD 20815 USA; ^10^ Present address: TEES‐AgriLife Center for Bioinformatics and Genomic Systems Engineering Texas A&M University 101 Gateway, Suite A College Station TX 77845 USA

**Keywords:** crossovers, deleterious SNPs, interstitial CO QTL, nested association mapping, polyploid wheat, recombination rate

## Abstract

Recombination affects the fate of alleles in populations by imposing constraints on the reshuffling of genetic information. Understanding the genetic basis of these constraints is critical for manipulating the recombination process to improve the resolution of genetic mapping, and reducing the negative effects of linkage drag and deleterious genetic load in breeding. Using sequence‐based genotyping of a wheat nested association mapping (NAM) population of 2,100 recombinant inbred lines created by crossing 29 diverse lines, we mapped QTL affecting the distribution and frequency of 102 000 crossovers (CO). Genome‐wide recombination rate variation was mostly defined by rare alleles with small effects together explaining up to 48.6% of variation. Most QTL were additive and showed predominantly *trans*‐acting effects. The QTL affecting the proximal COs also acted additively without increasing the frequency of distal COs. We showed that the regions with decreased recombination carry more single nucleotide polymorphisms (SNPs) with possible deleterious effects than the regions with a high recombination rate. Therefore, our study offers insights into the genetic basis of recombination rate variation in wheat and its effect on the distribution of deleterious SNPs across the genome. The identified *trans*‐acting additive QTL can be utilized to manipulate CO frequency and distribution in the large polyploid wheat genome opening the possibility to improve the efficiency of gene pyramiding and reducing the deleterious genetic load in the low‐recombining pericentromeric regions of chromosomes.

## Significance Statement

This study offers insights into the genetic basis of recombination rate variation in allopolyploid wheat and its effect on the distribution of deleterious SNPs across the genome. The identified *trans*‐acting additive recombination rate QTL can be utilized to manipulate crossover frequency and distribution in the large polyploid wheat genome opening the possibility to improve the efficiency of gene pyramiding and reducing the deleterious genetic load in the low recombining pericentromeric regions of chromosomes.

## Introduction

Besides playing a critical role in meiotic division, recombination is one of the major factors that influences the precision of gene mapping studies, and along with random drift and effective population size, defines the fate of genetic variation in populations by affecting the efficiency of selection acting on linked alleles. Recombination can break an allele's linkage with the genetic backgrounds that may harbor other alleles with either deleterious or beneficial effects, and increase the rate of selected allele fixation (Hill and Robertson, 1966; Comeron *et al*., 2008). Thereby, heterogeneity in the frequency of recombination events across the genome imposes different levels of constraint on the reshuffling of genetic variants in breeding and mapping populations, making the identification of the major determinants of recombination and the development of approaches for its modification important goals for the genetics of agricultural crops.

Intra‐species variability in the recombination rate observed for plants and animals (Dvorák and McGuire, 1981; Lynn *et al*., 2004; Esch *et al*., 2007; Pradillo *et al*., 2012; Bauer *et al*., 2013; Dreissig *et al*., 2015; Ziolkowski *et al*., 2017) was shown to be affected by both genes controlling meiotic division and the distribution of local genomic features and/or chromatin structure (Akhunov *et al*., 2003; Yandeau‐Nelson *et al*., 2006; Liu *et al*., 2009; Wijnker *et al*., 2013; Rodgers‐Melnick *et al*., 2015; Shilo *et al*., 2015; Melamed‐bessudo *et al*., 2016). Meiotically induced double‐stranded breaks (DSBs) are repaired through pathways resulting in either crossover (CO) or non‐crossover events, which can be detected when the interaction of the DSB region occurs between non‐sister chromatids (Mercier *et al*., 2015). Recent studies in model species, including Arabidopsis, demonstrated the feasibility of manipulating recombination by affecting meiotic genes in the CO pathways (Lieberman‐Lazarovich *et al*., 2013; Emmanuel *et al*., 2006; Higgins *et al*., 2008; Da Ines *et al*., 2013; Shilo *et al*., 2015; Crismani *et al*., 2012; Kirik *et al*., 2006; Wang *et al*., 2015; Colas *et al*., 2016). The ability to control the rate and distribution of recombination events has the potential to substantially accelerate the development of new varieties by allowing quick assembly of novel beneficial multi‐allelic complexes and by fixing desirable haplotypes in fewer generations. While these findings hold great promise for plant breeding, their practical applications are hindered by the limited understanding of the genetics of recombination rate control in specific crops that may differ in genomic organization, genome size, or ploidy level from those of the model species (Mercier *et al*., 2015). For example, some of the most important agricultural crops, like wheat, maize, or barley contain large regions of the genome comprised of transposable elements, preferentially located in the pericentromeric regions, in which recombination is severely suppressed (Akhunov *et al*., 2003; Saintenac *et al*., 2011; Choulet *et al*., 2014; Wingen *et al*., 2017). Due to the reduced efficiency of background selection, these genomes were shown to be enriched for SNPs with possible deleterious effects (Mezmouk and Ross‐Ibarra, 2014; Liu *et al*., 2017). The reduction of this deleterious genetic load is one of the possible strategies to accelerate crop improvement. In addition, reduced recombination also diminishes the efficiency of selection for favorable alleles when they are linked with deleterious variants. Therefore, the identification of genetic factors that can shift CO distribution toward the pericentromeric chromosomal regions would be important for making all genomic regions equally accessible for selection.

The large genome size, polyploidy, and the availability of novel genomic and genetic resources make wheat a tractable system for studying multiple aspects of meiotic recombination genetics in crops with complex genomes. Here, we developed and densely genotyped a wheat NAM population to study the genetic architecture of genome‐wide recombination rate variation and to identify QTL controlling CO distribution and frequency. Using these datasets, we investigated various aspects of the genetics of recombination rate control in an allopolyploid crop, and studied the effects of genome‐wide recombination rate variation on the distribution of deleterious SNPs in the wheat genomes. We showed that the recombination rate variation along the chromosomes and among the genomes affects the distribution of deleterious genetic load. Using the identified QTL, we demonstrated that the distribution of recombination events along the centromere–telomere axis of chromosomes is genetically controlled by multiple loci with additive effects. Our study provides valuable information for understanding the genetic architecture of recombination rate in an allopolyploid crop, and lays the foundation for developing approaches to manipulate recombination rate distribution across the genome.

## Results

### Genotyping NAM population

**Figure 1:**
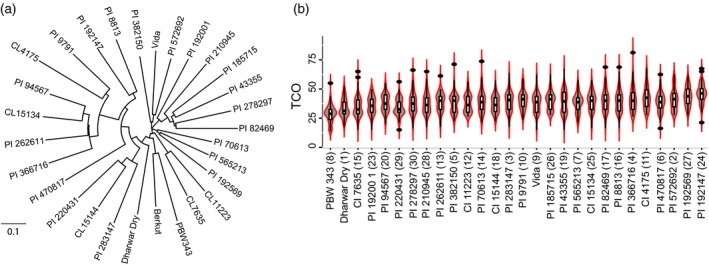
Recombination rate variation in the wheat NAM population. (a) Phylogenetic tree of wheat accessions used to develop the wheat NAM population using the pairwise divergence estimates of genome‐wide SNPs. (b) Violin plot showing the distribution of total crossovers (TCO) for each NAM family. NAM family distinction is the represented by number in parenthesis after the name of the corresponding NAM founding parent.

To develop a nested association mapping population, a diverse set of 29 wheat accessions including four cultivars and 25 landraces (henceforth, founder lines) was selected to represent both the genetic and geographic diversity of wheat (Figure 1a and Table S1). The former was assessed using the 9K iSelect assay genotyping data generated for a large worldwide collection of wheat accessions (Cavanagh *et al*., 2013). Additional high‐density SNP and InDel data was generated for founder lines using the wheat exome capture assay (WEC) (Jordan *et al*., 2015) and the 420K Axiom array. The variant calling in the WEC dataset mapped to the W7984 wheat reference genome (Chapman *et al*., 2015) resulted in 638 045 SNPs and 63 022 bi‐allelic insertion–deletion (InDel) polymorphisms (Tables S2 and S3). Genotyping using the 420K Axiom array yielded 149 685 bi‐allelic polymorphisms (Table S4), 114 590 of which are mapped to the W7984 reference genome. In total, using these genotyping approaches we identified over 800 000 bi‐allelic polymorphisms segregating within the NAM population. Consistent with previous results (Wang *et al*., 2014; Jordan *et al*., 2015), the A and B genomes were more polymorphic than the D genome (Figure S1a). The frequency spectrum of variants detected using the Axiom array was shifted toward common alleles consistent with ascertainment bias inherent in genotyping arrays (Figure S1b). No allele frequency shift was found for the WEC variants except for the depletion of alleles among unique variants (MAF = 1/29) due to filtering for alleles present in at least two founders (see Experimental procedures).

In addition, the Recombinant inbred lines (RILs) and founders were genotyped using the 90K SNP array (Wang *et al*., 2014) and by genotyping by sequencing (GBS) (Saintenac *et al*., 2013). Nearly 4 billion Illumina reads were generated for all founder lines and NAM RILs, with an average of 1.63 million reads per RIL (Table S5), and 3 million reads per founder. Utilizing different SNP calling pipelines, we detected 164 668 unique GBS SNP markers across 28 populations with the majority of SNPs being family‐specific (Tables S6 and S7 and Figure S2a, b). In addition to SNPs, a custom pipeline (Saintenac *et al*., 2013) was used to call presence–absence variants (PAV) in GBS data, resulting in 331 090 PAVs (Tables S6 and S7), 64% of which were family‐specific. The 90K SNP array resulted in 57 687 polymorphic markers in the NAM population (Tables S8 and S9). Both Axiom and 90K SNP array datasets showed an enrichment for common alleles (Figures S1b and S2b), thereby providing the opportunity to assess the allelic effects across multiple genetic backgrounds. By combining sequence‐ and array‐based SNPs, and PAV, an average of 50,143 markers were placed onto family‐specific genetic maps (Figure S3 and Table S10).

### Distribution of COs in family‐specific genetic maps

The relatively small number of recombination events in bi‐parental mapping populations and high‐density genotyping using the array‐ and sequence‐based approaches results in highly redundant marker data per recombination bin defined by the recombination breakpoints on the chromosomes. Genotyping errors and missing data in these large datasets provide substantial challenges for map construction algorithms (Ronin *et al*., 2017). Here, for genetic map construction, the marker redundancy was removed by pre‐processing genotyping data using the clustering algorithm implemented in *Multipoint* software (for details and rationale see Experimental procedures). A representative set of markers for each population was used for constructing 28 family‐specific genetic maps (Table S11). The average map size was 1,286 cM for the A genome (0.23 cM per Mb), 1,178 cM for the B genome (0.19 cM per Mb), and 855 cM for the D genome (0.17 cM per Mb) (Table S12). The map lengths of the D genome chromosomes were consistently shorter than those in the A and B genomes. One of possible explanations for these results can be an elevated CO rate in more divergent genomes consistent with the positive effect of inter‐homolog polymorphism on COs observed in *Arabidopsis* (Ziolkowski *et al*., 2015). We investigated the effect of genetic diversity, which is lower in the D genome compared with that in the A and B genomes, on the estimates of map length and recombination rate in the D genome. For this purpose, we generated sets of A and B genome markers by randomly selecting the same number of SNPs that were genotyped in the D genome of one of the NAM families (e.g. NAM19). The lengths of genetic maps for the A, B and D genomes developed using the NAM19 were 1240.5 cM (0.22 cM/Mb), 1160.0 cM (0.19 cM/Mb), and 798.8 cM (0.16 cM/Mb), respectively. Based on 15 genetic maps constructed using thinned SNP datasets sampled from the NAM19 family, the average lengths of genetic maps in the A and B genomes were 1110.8 ± 15.5 cM (0.19 cM/Mb), and 987.3 ± 18.1 cM (0.16 cM/Mb). The estimates of recombination rate (cM/Mb) using these simulated maps were close to the estimates obtained for the D genome in the NAM19 family (Table S12). Additionally, the average length of the simulated A and B genome maps (2,098.1 cM) was lower than the map lengths for the A and B genomes in NAM19 (2400.5 cM) or tetraploid durum wheat (2464 cM) (Maccaferri *et al*., 2015). These results suggested that the underestimation of double COs due to low marker density and reduced genetic diversity in the D genome can contribute to differences observed in the map length estimates between the A/B genomes and the D genome.

Meiotic COs were detected using the family‐specific maps as a change in the phase of at least two parental alleles for each RIL in each NAM family (for rationale see Experimental procedures), resulting in more than 102 000 recombination events in the NAM population (Table S13). As a measure of recombination rate, we used the total number of COs (TCO) for each individual RIL (Table S14). We found variation within and between NAM families for TCO (Figure 1b) with the overall mean of the entire population 48.1 ± 0.18, consistent with the estimates obtained in wheat by immunolocalization of MLH1, which marks class I COs (Martín *et al*., 2014). The NAM8 family contained the lowest TCO with a mean of 37.7 ± 0.8, and the NAM24 family exhibited the highest TCO with the mean of 55.7 ± 0.9 (Figure 1(b) and Table S15). It should be noted that due to heterozygosity in the initial generations of inbreeding, some COs observed in RILs can represent different meiotic events. In addition, the recombination rate modifiers can change between heterozygous and homozygous states during inbreeding, which may have a confounding effect on the estimates of recombination. These confounding factors, however, would most likely result in an underestimation of the number of QTL due to reduced effect of recombination modifiers maintained in heterozygous state. Thus, very likely in our families the number of QTL harboring recombination modifiers with moderate or weak effects are underestimated.

**Figure 2:**
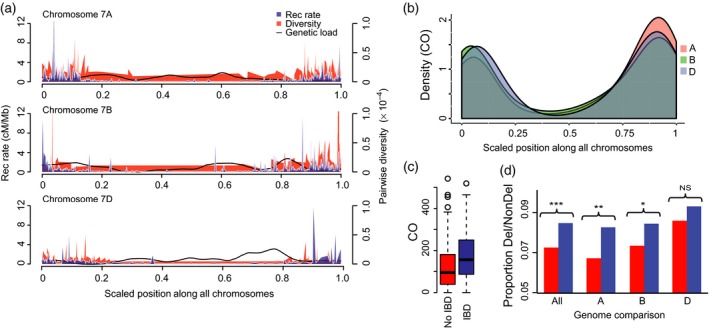
Distribution of COs, recombination rate, and deleterious load across the wheat genome. (a) Distribution of recombination rate (cM/Mb), genetic diversity, and deleterious allele density across the chromosomes group 7. Deleterious allele load is the ratio (right *y*‐axis) of number of potentially deleterious SNPs to the total number of coding region SNPs for each genetic bin on the scaled chromosome length. Average recombination rate (cM/Mb) across all 28 families is shown for 2 Mb windows stepping 1 Mb a time on a scaled *x*‐axis for comparisons of all chromosomes. Diversity is the average pairwise diversity of across all families. (b) Distribution of crossovers along the wheat chromosomes in the A, B and D genomes. The positions of each breakpoint along each chromosome was expressed in the scale from 0 to 1 and combined for each genome. (c) Comparison of the total number of COs between the genomic regions of the NAM founders that share (red) or do not share (blue) IBD with the common parent Berkut. The difference was statistically significant at *P*‐value ≤ 10^−16^ (Mann–Whitney *U*‐test). (d) Proportion of deleterious to non‐deleterious SNP alleles across each genome. High‐recombining region estimates are shown in red; the estimates for the rest of the genome are shown in blue. Stars above the plot represent statistical significance within each genome comparison: ***P < 10^−5^; **P < 10^−4^; *P = 0.01.

Meiotic recombination was distributed non‐uniformly along the chromosomes (Akhunov *et al*., 2003; Saintenac *et al*., 2011; Rodgers‐Melnick *et al*., 2015) and varied among the wheat genomes (Figures 2a, b, S4–10 lower panels and S11 and Table S13), with the distal one‐third of the chromosomal arms harboring three times more COs than the pericentromeric regions (χ^2^‐test <10^−4^) (Figure 2b). Using the improved estimates of meiotic recombination rate in this study, we assessed its relationship with the historic recombination events measured by calculating linkage disequilibrium (LD) for SNPs in the population of the founding lines. A significantly negative correlation (*r*_*p*_^2^ = −0.17, *P*‐value < 10^−13^; Figures S4–10 lower panels) between LD and the meiotic recombination suggests that the historic decline in inter‐variant linkage in the founders is associated with the regions also showing high recombination rates in the bi‐parental crosses.

### The frequency of meiotic COs and founder divergence

To investigate the effect of genome‐wide founder divergence on the frequency of meiotic recombination, we utilized polymorphic GBS tags mapped to the reference genome and showing the presence of PAV and SNP variation (Table S16). Even though PAVs accounted for 57% of the variants, only 36% of PAVs could be mapped to the reference, compared with 50% of the SNPs, suggesting that nearly two‐thirds of the PAVs have no match in the reference genome, indicating either sequence or structural divergence of the NAM founders from the reference genome. On average, we detected 3 PAVs per Mb, and 27 PAVs per recombination bin on the genetic map (Table S17).

The genome‐wide divergence of the founder lines from the common parent Berkut explained a relatively small proportion of inter‐family recombination rate variation measured as the TCO family mean (*R* ^2^ = 0.06 for PAV, *P*‐value = 0.20 and *R* ^2^ = 0.02 for SNPs, *P*‐value = 0.48, Figure S12). However, in the regions in which founder lines were identical‐by‐descent (IBD) to the common parent Berkut, we found significantly more COs (non‐parametric *U*‐test *P*‐value ≤ 10^−16^) than in the regions showing no evidence of IBD (Figure 2c). This result was consistent with a study showing a negative effect of local sequence divergence between wheat chromosomes on local recombination rate (Saintenac *et al*., 2011).

### Distribution of predicted deleterious SNPs across and among the wheat genomes

Using the estimates of recombination rate variation, we investigated its effect on the genome‐wide distribution of non‐synonymous SNPs with tentatively deleterious effects on protein function. It was hypothesized that deleterious genetic load is effectively removed from populations in regions of high meiotic recombination (Hartfield and Glémin, 2014). However, it is not known how gene redundancy resulting from polyploidy would affect the genomic distribution and frequency of predicted deleterious alleles in the populations of a polyploid species. In wheat, we identified 1168, 1594 and 437 predicted highly deleterious SNPs, in the A, B and D genomes, respectively. Their proportions relative to predicted non‐deleterious alleles were higher in the D genome (0.089) than in the A and B genomes (0.076 and 0.081, respectively). However, only in the A–D genome comparison the difference was statistically significant (Fisher's exact test, *P = *0.01). Considering the entire dataset of predicted deleterious and non‐deleterious SNPs, we found a significant reduction in the proportion of deleterious SNPs in the high‐recombining regions compared with the rest of the genome (*P = *7.8 × 10^−5^; Figure 2d). This reduction of genetic load in the high‐recombining chromosomal regions was consistent across all three wheat genomes, but only statistically significant in the A and B genomes (A genome, *P = *6.0 × 10^−4^, B genome, *P = *0.01, and D genome *P = *0.20, Figure 2d). A lower frequency of deleterious mutations was previously observed in the regions of high recombination in many species supporting a role for recombination in ‘purging’ deleterious alleles (Charlesworth and Charlesworth, 1998).

### Recombination rate QTL mapping in the NAM families

#### QTL mapping

QTL mapping in individual NAM families detected 40 QTL for TCO at a genome‐wide significance threshold of 0.05 (Table S18). Markers mapped to the reference genome were used to infer the 2‐LOD support interval boundaries of these 40 QTL regions (Table S18), resulting in 27 non‐overlapping, unique regions of the genome. TCO QTL were detected in 22 of the 28 families, and are located on 16 of the wheat chromosomes, mostly in the A and B genomes (Table 1). The average effect size was 3.36 ± 0.23 CO, which is a 7.0% change compared with the mean (Table 1). The largest effects were detected in the NAM4 family in which the Berkut allele on the long arm of chromosome 7B increased the recombination by 9.2 COs, and another region on the same chromosome decreased recombination by 9.1 COs.

**Table 1:** The results of TCO QTL mapping in individual NAM families

Trait	Genome	Number of QTL	Average effect^a^	Effect size^b^
	Whole genome	40	3.36	7.0%
TCO	A	18	3.16	6.6%
	B	18	3.61	7.5%
	D	4	3.17	6.6%

a Average effect size is the magnitude of effect of the favorable allele.

b Effect size is the percent effect with respect to the overall mean for TCO.

Chromosome 7B harbored the most QTL of any chromosome, and the region at 51.2–59.2 cM was found in three separate families (Figure S10). Overall, eight of 27 unique TCO QTL regions (30%) were identified in more than one family, and three regions in three families (Table S19). In three of these overlapping QTL regions, the common parent Berkut's allele affected TCO positively in all families in which these QTL were detected, and in one case on 4B, Berkut's allele negatively affected TCO in both families. Whereas at the remaining overlapping QTL, the direction of Berkut's allele effect was inconsistent between the families, suggesting the existence of allelic series of genes. For example, at the QTL located on chromosome 6A (88.0–98.3 cM), Berkut's allele had a positive effect in family NAM27, and a negative effect in family NAM25. Similar allelic series, with both positive and negative effects, were observed in maize (Buckler *et al*., 2009). Overall, Berkut alleles in these eight QTL regions positively affected TCO in 13 families (65% of families) (Table S19), and other parents contributed positive alleles in seven families (35% of families).

Out of the 40 detected QTL, 13 (33%) remained significant even at the Bonferroni‐corrected threshold of 0.0018 (0.05/28) (Table S18). Two TCO QTL were mapped in two families at both thresholds, chromosome 4A mapped in families NAM19 and NAM30 (Figure S7), and a QTL on chromosome 7B at 94–101 cM mapped in families NAM7 and NAM4 (Figure S10). However, due to the conservative nature of Bonferroni correction, it appeared that many valid QTL did not pass this strict significance threshold. For example, a TCO QTL mapped to chromosome 4B in families NAM19 and NAM30 was detected only in one family at 0.0018 threshold. Similarly, a TCO QTL mapped to chromosome 6B in three different families, was not detected in any NAM family at the stricter threshold (Table S18 and Figure S9).

#### Additivity of bi‐parental family QTL

**Figure 3:**
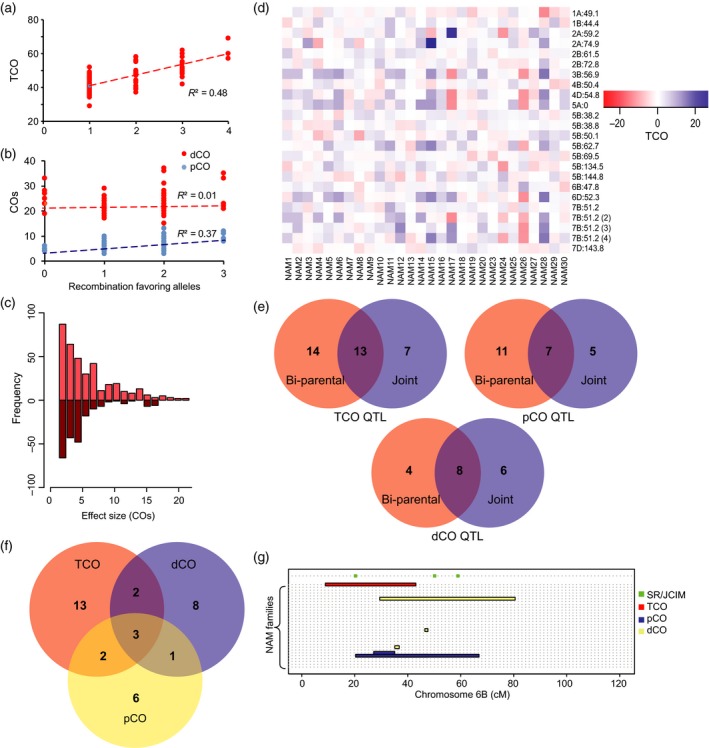
Recombination rate QTL mapping. (a) The number of recombination increasing alleles in the RILs correlates positively with the number of TCOs (*R* ^2 ^= 0.48) for family NAM30. (b) The number of positive alleles at pCO QTL (blue) correlates positively with the interstitial COs (*R* ^*2*^ = 0.36) and shows no correlation (*R* ^*2*^ = 0.01) with dCOs in the distal (red) regions for family NAM29. (c) The frequency of additive alleles with different effect sizes for the TCO QTL. The majority of TCO QTL alleles showed small effect. (d) Magnitude of effect sizes relative to Berkut allele for TCO QTL identified using the SR and JCIM approaches. Positive values indicate that the Berkut allele had a positive effect on recombination. Blue hues represent positive effects, i.e. Berkut allele favors increased recombination, the red hues represent negative effects, i.e. Berkut allele suppresses recombination. Intensity of effect ranges with the darker shades of the boxes (e) Venn diagram depicting overlap of bi‐parental mapping versus joint mapping for TCO, pCO, and dCO. (f) Venn diagram showing overlap of the joint mapped QTL regions detected for the TCO, pCO, and dCO. (g) Summary of QTL mapping results in 28 NAM families for chromosome 6B. Each family is represented on the *y*‐axis with horizontal dashed lines, starting with NAM1 up to NAM30. Red, blue, and yellow bars represent QTL regions for TCO, pCO, and dCO, respectively, found in bi‐parental mapping populations. Green squares represent significant SR and JCIM regions

In the NAM families with more than one recombination QTL, we estimated the effects of multiple positive alleles on TCO (Figure S13). There are 15 NAM families that contained more than one QTL. Only one family did not exhibit a significant correlation between the number of recombination favoring alleles and the total amount of recombination events. Over all families, the number of positive alleles explained up to 48% of the variation in recombination rate (Figure 3a and Table S20). In the NAM30 family, four QTL were detected, and we observed a phenotypic difference of more than 12 COs/genome between the RILs carrying only one and all four recombination favorable alleles (Figures 3a and S13).

#### Cis‐ *and* trans‐*acting QTL*

To discriminate between QTL that affect recombination locally (*cis*‐effect) from those that have global effects (*trans*‐effect), we removed recombination data for the chromosome harboring that particular QTL from the calculations of the TCO, and repeated QTL mapping analyses (Esch *et al*., 2007). By performing this procedure for each of the 40 TCO QTL, we re‐discovered 28, suggesting that 70% of the TCO QTL exhibited *trans*‐acting effects (Table S21).

#### QTL affecting the distribution of recombination along the telomere‐centromere axis

In most organisms including wheat (Akhunov *et al*., 2003; Lynn *et al*., 2004; Saintenac *et al*., 2011; Rodgers‐Melnick *et al*., 2015), COs are more likely to occur on the distal ends of the chromosomes, as opposed to the pericentromeric regions. Our data are consistent with this phenomenon (Figures 2a, b and S11), in which 75% of the observed COs are located in the distal ends of the chromosomes. However, it is unknown whether the distribution of COs along the chromosomes can be controlled genetically or are defined by the distribution of other genomic features.

Using COs grouped by the location of breakpoints in the distal (1/3 of chromosome arm temini) or pericentromeric (2/3 of chromosome arm adjacent to the centromere) regions, we identified 24 QTL for pericentromeric COs (henceforth, pCOs) with an average effect size of 1.2 ± 0.05 COs, and 15 QTL for distal COs (henceforth, dCOs) (Table S22), with an average effect of 2.3 ± 0.12 COs. Only one of these pCO QTL overlapped with the original TCO QTL detected within the same family utilizing whole chromosome data. Over 80% of dCO QTL regions overlapped with the TCO QTL, consistent with 75% of the observed recombination events coming from the distal ends. We were able to partition the local and global effects on recombination of the pCO and dCO similarly as for TCO. For the pCO QTL, 13 of 24 (56%) were *trans*‐acting, suggesting that over half the pCO QTL affect recombination globally. Similarly, 60% (9 of 15) dCO affecting distal CO were found to possess *trans*‐effects, consistent with number of *trans*‐acting TCO QTL.

For the four families with more than one *trans*‐acting QTL affecting pCOs, we investigated the effect of variation in the number of positive alleles on the frequency of COs in the distal and pericentromeric regions. The positive alleles acted additively and explained, on average, 29% of variation in the pericentromeric COs, while only 1.7 and 12% of variation in the distal COs and TCO, respectively (Table 2, Figure 3b, S14). For example, in family NAM29, the RILs carrying three favorable alleles had an additional six pericentromeric COs compared with the RILs carrying no favorable alleles (Table 2, Figure 3b, S14). The observation that pCO and TCO QTL regions show little overlap, and that combining additive *trans*‐acting pCO QTL increases COs in the pericentromeric region without affecting distal COs (Table 2), suggests the possibility that different genetic factors could contribute to the distribution of recombination events between the pericentromeric and distal chromosomal regions. These results also suggest that by combining the alleles of *trans*‐effect QTL it should be possible to direct recombination to the pericentromeric regions of wheat chromosomes.

**Table 2:** The additive effect of positive alleles at the *trans*‐acting QTL for pericentromeric recombination rate on the number of crossovers (COs) in the distal and pericentromeric regions of chromosomes

Family	Proportion of CO variance (R^2^) explained by the number of positive alleles at the pericentromeric recombination rate QTL in RILs^a^			Maximum effect size^b^	Number of positive alleles/RIL
	pCO	dCO	TCO		
NAM6	0.28	1.0 × 10^−4^	0.08	4.0	2
NAM8	0.30	9.0 × 10^−4^	0.10	3.6	2
NAM10	0.20	5.6 × 10^−2^	0.18	4.6	3
NAM29	0.37	1.1 × 10^−2^	0.15	5.9	3

a Statically significant at *P*‐value ≤ 0.05.

b The maximum difference in the number of COs between RILs with 0 and maximal number of positive alleles at the QTL for pericentromeric recombination rate.

### Joint mapping of recombination QTL in the NAM population

In general, the NAM design allows for mapping QTL across families, while adjusting for the common parent family structure. The NAM design increases the resolution and power of QTL detection over traditional bi‐parental QTL mapping, especially for alleles that are shared across families. We took advantage of the NAM design to perform joint stepwise regression (SR) and joint inclusive composite interval mapping (JICIM) (Buckler *et al*., 2009) of recombination QTL (Tables [Link], [Link], [Link]). We detected 15 major SR QTL (Table S23) that explain more than 48% of the variance in TCO in the NAM population, and found evidence for nine additional minor JICIM QTL regions (Table S24). The average allele effects of the TCO QTL mapped using joint mapping across all families was 3.94 ± 0.18 COs (Figure 3c, d and Tables S24 and S25), an 8.2% change in TCO rate relative to the average. There were more QTL alleles increasing CO rate than decreasing it with respect to the common parent allele (Figure 3c). The largest effects detected were found on chromosome 7B QTL, consistent with the bi‐parental mapping results. Of the 24 joint mapping QTL detected, two SR loci located within 1 cM of each other were detected on chromosome 5B, while the region on chromosome 7B at 51.19 cM possessed three separate JICIM QTL intervals and one SR QTL, thus identifying 20 unique, significant regions in the genome (Figures S4–S10 upper panels). Together, joint mapping found evidence of QTL located on 13 chromosomes that affect COs in the NAM population (Figures 3d and S4–S10 upper panels). Nine of the 14 unique SR QTL regions (64%) and four of the seven unique JICIM QTL regions (57%) fall within the 27 unique TCO QTL regions identified in the single‐family analyses at the 0.05 significance threshold (Table S18). At the more strict Bonferroni‐corrected significance threshold (0.0018), QTL detected in single‐family analyses showed an even smaller overlap with QTL detected using joint mapping approaches. Out of 10 QTL regions mapped by single‐family analyses, only three QTL were identified by SR and two QTL were identified by JICIM. These results further corroborate our earlier conclusion (see single‐family QTL mapping results) that the Bonferroni‐corrected significance threshold likely results in an increased false‐negative rate. Overall, 13 of the 20 (65%) QTL regions detected by joint mapping overlapped with the 27 unique single‐family QTL regions. Likewise, 48% (13 of 27) of unique regions identified by single‐family QTL analysis are detected by joint mapping approaches (Figure 3e). The modest overlap between the QTL detected in the single‐family analyses and joint mapping observed in our study was previously reported in the maize NAM population (Ogut *et al*., 2015). It was suggested that the design of the NAM population allows for detecting more common QTL with minor effects, it also may have reduced power of detecting rare QTL unique to each family (Ogut *et al*., 2015), suggesting the complementary nature of using multiple mapping strategies to dissect QTL affecting traits.

In terms of numbers of unique QTL regions affecting traits, and the magnitude of effect sizes for QTL, joint mapping and single‐family mapping approaches were similar, but the mapping resolution was increased using joint mapping, as most QTL fall to a single genetic position or adjacent intervals on the reference map. The NAM design allowed for assessing the effect of the Berkut alleles in different genetic backgrounds. Across all QTL and all families, joint mapping detected that ~60% of Berkut alleles had positive effect on CO rate (Figures 3c, d and S15). Thereby, 40% of recombination favoring alleles were contributed by the other parents. Similar to findings from the maize flowering time QTL mapping study (Buckler *et al*., 2009), we found that the direction of QTL effects was not consistent across families indicating the presence of allelic series at QTL (Figure 3d and Tables S24 and S25). The distribution of recombination allele effect sizes was similar to that observed for quantitative traits (Buckler *et al*., 2009), in which the majority of effect sizes observed are small, and major effect alleles are more rare in the population (Figures 3c and S15). However, in comparison with QTL identified for various quantitative traits in maize (Buckler *et al*., 2009; Brown *et al*., 2011), recombination QTL in wheat were present at a low frequency (Figure S16).

Stepwise regression and JICIM mapping for the pCO trait detected 10 and five QTL, respectively (Tables [Link], [Link], [Link]), with an average effect of 0.77 ± 0.04 COs (Figure S15). The region on chromosome 2B from 59 to 61 cM was defined by 2 JICIM intervals and also contained one locus detected by SR; the region on chromosome 3A at 20.49 cM was detected by both JICIM and SR mapping. Together these QTL were mapped to 12 unique genomic regions and explained 38.9% of variation in pCOs. Seven of these 12 pCO QTL regions (58%) were also detected in the bi‐parental mapping families for pCO QTL (Figure 3e), and five of these 12 (42%) co‐localized with the TCO QTL detected by joint mapping (Figure 3f).

Joint mapping of dCOs detected six SR QTL and nine minor JICIM QTL (Tables [Link], [Link], [Link]), with an average effect size of 1.88 ± 0.09 COs (Figure S15), together explaining 41% of the observed trait variation. The region on chromosome 1B at 44.4 cM was detected in two JICIM intervals, giving 14 unique regions affecting dCOs. Eight of these 14 unique dCO QTL regions (57%) overlapped with dCO QTL regions detected within the NAM families; eight of 12 (67%) unique single‐family QTL regions for dCO overlapped with the dCO QTL regions detected by joint mapping (Figure 3e). Five of the joint mapping dCO QTL co‐localized with TCO QTL detected by joint mapping (Figure 3f). Only four QTL regions for dCO and pCO detected by joint mapping overlapped, suggesting some uncoupling of genetic factors affecting recombination events in the distal and proximal regions of wheat chromosomes.

### Refined recombination rate QTL regions contain conserved meiotic genes

Using a conservative approach to define genomic intervals affecting recombination rate mapped in the individual NAM families (detected in at least two families), we find eight regions affecting TCO, six regions affecting pCO, and three regions affecting dCOs (Table S19). For TCO, all four regions in which the parental effect estimates were in the same direction, we also detected the region or a region in close proximity using joint mapping, confirming its power to detect regions in which alleles are shared across families. The four regions not detected include the region on chromosome 4A (111 cM) harboring QTL with opposite allelic effects in three families. In NAM30, the Berkut allele had a positive effect, while in NAM19 and NAM7, the Berkut allele had a negative effect of similar magnitude. For QTL on chromosome 7B (94–100 cM), also detected in three families, NAM 4 had an allele with large negative effect, while NAM6 and NAM7 have positive effect allele with smaller effect sizes. The QTL region on chromosome 6A (88–98 cM), also had alleles with opposing signs in NAM25 and NAM27, as did the QTL region on chromosome 7A (0–30 cM) in NAM20 and NAM26 that have the same size effects in different directions.

Incorporating the joint mapping results with the bi‐parental QTL mapping, we were able to narrow the candidate regions affecting variation in recombination to 14 regions spanning 12 chromosomes (Table S26), supporting the idea of multiple loci affecting recombination within the NAM population. The most consistently detected regions included the regions of chromosome 1A at 41–49 cM, chromosome 2B at 59–61 cM, and on chromosome 6B at 43–48 cM, which were detected by joint mapping for each component of TCO (Figure 3g). Using the joint mapping approach, we also detected two regions for both TCO and dCOs traits on chromosome 1B at 44 cM, and on chromosome 4B at 50–58 cM.

Candidate gene searches in these 14 regions included orthologs of many genes previously shown to effect recombination in wheat and other species, including well‐conserved meiotic genes, such as, *SPO11‐2*,* RAD51*,* DMC1*,* FANCM*,* RAD54*,* MutS*,* RECQ4*,* TOP3*α, *HEI10*,* ZYP1*, and *PRD3* (Lawrence *et al*., 2017) (Table S27). In some cases, we uncover syntenic regions of chromosomes that harbor homoeologs of these conserved recombination genes. For instance, we find the syntenic regions of 2A and 2B at 59.2 cM and 59.2 cM respectively, in which both the A and B homologs of *MSH3* and *ZYP1* are located. Similar cases are found on chromosomes 6A and 6B, in which wheat homologs of *HEI10* are detected at 50.2 cM and 47.8 cM, respectively. In addition to these candidates, we find regions of interest that contain other multiple homoeologs of *DMC1*,* RecQ helicase*, and *RAD23b* (Table S28) in more than one wheat genome. Although, these results suggest the possibility that variation at these genes’ loci may contribute to natural variation in recombination rate among wheat accessions, further studies aimed at confirming the role of these wheat homologs in controlling CO frequency are needed. In addition to QTL harboring conserved meiotic genes, there were recombination QTL regions lacking *a priori* candidate genes, which suggests that other uncharacterized determinants of recombination rate variation are present in the population.

## Discussion

Here, we developed a spring wheat NAM population and used it to dissect the genetic architecture of recombination rate variation, and to study the relationship between recombination, genetic variation, and deleterious SNPs in an allopolyploid crop with a large, highly repetitive genome. This NAM population further expands the number of multi‐parent mapping populations available to the wheat community for studying the genetic architecture of complex traits (Cavanagh *et al*., 2013; Bajgain *et al*., 2016; Gardner *et al*., 2016; Wingen *et al*., 2017).

The uneven distribution of recombination along the wheat chromosomes was shown to have a profound effect on the distribution of genetic diversity (Akhunov *et al*., 2010; Jordan *et al*., 2015), especially on the distribution of non‐synonymous mutations with potentially deleterious effects on protein function. As these deleterious alleles may affect agronomic traits, they have attracted attention as potential targets for trait improvement (Yang *et al*., 2017). While reduced deleterious allele load in the high‐recombining regions was demonstrated in several diploid crops, such as maize, rice, and grape (Mezmouk and Ross‐Ibarra, 2014; Liu *et al*., 2017; Zhou *et al*., 2017), it remained unclear how the distribution of deleterious alleles is affected by polyploidy and the resulting relaxation of selection constraints on duplicated genes. The finding that all three wheat genomes show similar patterns of deleterious allele distribution along chromosomes, suggests that polyploidy does not affect the negative correlation between recombination rate and deleterious alleles previously found in diploid plants (Mezmouk and Ross‐Ibarra, 2014; Liu *et al*., 2017). The deleterious alleles in allopolyploid wheat are likely still under selection that is more effective in the regions of high recombination, and tends to favor the retention of functional duplicated homoeologs. These results are consistent with earlier observations showing that many genes in wheat are transcriptionally active and likely functional (Akhunova *et al*., 2010; Pfeifer *et al*., 2014).

We found no significant differences in the proportion of deleterious to non‐deleterious alleles in the high and low‐recombining regions of the D genome compared with that in the A and B genomes. One of the possible explanations is the recent population bottleneck associated with the recent whole‐genome merger between tetraploid wheat and the ancestor of the wheat D genome about 10 000 years ago, and the subsequent gene flow between wheat and tetraploid ancestral populations (Dvorák *et al*., 2006; Jordan *et al*., 2015). This gene flow was suggested to play a significant role in restoring the diversity of the A and B genomes, possibly increasing both the effective population size and the efficiency of selection against deleterious alleles. The inability of wheat – *Ae*. *tauschii* hybrids to produce fertile progeny was proposed as one of the main reasons for reduced diversity in the wheat D genome (Akhunov *et al*., 2010) that likely resulted in increased genetic drift and reduced the efficiency of selection.

We applied single‐family, and joint‐linkage mapping in the wheat NAM population to gain insights into the genetic architecture of recombination rate variation in an allopolyploid crop with a large, highly repetitive genome. Irrespective of the applied thresholds, we found that 48% of QTL mapped in single‐family analyses overlapped with QTL mapped using the joint mapping approach. These results were consistent with the results of a recent study performed using the maize NAM population (Ogut *et al*., 2015), in which it was shown that the proportion of overlapping QTL identified by single‐family analysis and by joint mapping can vary for different traits and often showed small overlap. For example, between 36 and 10 QTL for oil content detected using single‐family and joint mapping, respectively, only four (11%) overlapped. Similarly, between the 31 single‐family and nine joint mapping QTL for days to anthesis trait, only six (24%) overlapped. The differences in the number of QTL mapped using different approaches were attributed to the lower power of the joint mapping approach to detect rare QTL compared with the single‐family analysis approach (Ogut *et al*., 2015). This study and our results suggest that the usage of both single‐family and joint mapping approaches with NAM populations might provide a more comprehensive view of the traits’ genetic architecture and help to identify rare, family‐specific QTL of biological interest.

The NAM design allowed us to assess the allelic effects of common parent alleles across different genetic backgrounds. While 60% of the Berkut alleles showed a positive effect on CO rate in the NAM population, the direction of the change was not consistent across families, indicating the dominance of the alternative parent's allele at nearly 40% of loci. Similar changes in the direction of the common parent allele effects found in the maize NAM population for flowering time QTL (Buckler *et al*., 2009), and were attributed to allelic series of genes that exist at a limited number of QTL. In wheat, allelic variation at homoeologous gene loci with redundant function might also have a confounding effect on the direction of the allelic affect at recombination QTL. While the low resolution of mapping does not allow us to precisely identify gene homoeologs, the existence of QTL at the homoeologous regions of chromosomes suggests the possibility that different gene homoeologs in different lines might control meiotic recombination in wheat. Further studies focusing on functional validation of meiotic gene homoeologs will be required in the future to test this hypothesis.

Within our *trans*‐acting QTL regions, we find candidate genes known for their involvement in meiotic recombination, suggesting that these genes may contribute to the genome‐wide recombination rate variation among the NAM founders. However, the lack of known meiotic candidate genes in some *trans*‐acting QTL regions suggests that other previously uncharacterized genetic factors or genes may affect variation in recombination rate in wheat.

The frequency distribution of alleles with different effect sizes in the population was similar to that observed for the majority of quantitative traits (Buckler *et al*., 2009; Brown *et al*., 2011), suggesting that recombination rate variation among wheat lines mostly results from variation in a number of alleles with small rather than large effects. However, in contrast to other quantitative traits in maize (Buckler *et al*., 2009; Brown *et al*., 2011), based on joint‐linkage mapping, the majority of alleles affecting recombination rate were family‐specific with only 30% of alleles being shared across the NAM families, suggesting that multiple founder‐specific genetic factors might define recombination rate variation in wheat. The additivity of their effects suggests the possibility of increasing recombination rate in wheat by pyramiding multiple alleles of these QTL. These alleles are of potential value for crop improvement, as it was suggested that the selection for increased recombination rate combined with selection for the traits of interest (Hill and Robertson, 1966; Mcclosky and Tanksley, 2013) may accelerate the rate of genetic gain.

Increasing recombination in the identified *trans*‐acting pericentromeric chromosomal regions (Akhunov *et al*., 2003; Saintenac *et al*., 2011) has the potential to substantially increase the efficiency of selection for favorable allelic variants located in these regions. Moreover, the results in our study suggest that increased recombination in the pericentromeric regions might also contribute to reducing the deleterious genetic load in these regions. Recently, the possibility of increasing the interstitial CO rate by increasing temperature (Higgins *et al*., 2012) was demonstrated in barley. Our results provided evidence for the existence of *trans*‐genetic factors, which act additively to promote COs in the pericentromeric regions without the concomitant increase in the number of COs in the distal regions. Combining the allelic variants of these QTL provides an alternative path to increasing the interstitial CO rate.

## Conclusions

Our study suggests that the QTL affecting recombination rate and distribution mapped using the wheat NAM population can be exploited for manipulating CO frequency. By combining positively or negatively *trans*‐acting QTL, genome‐wide recombination can be increased to break linkages or decreased to quickly fix beneficial haplotypes, respectively. Even in the genetically redundant polyploid genome, the distribution of crossovers was shown to be one of the main factors affecting the distribution of potentially detrimental allelic variants resulting in their accumulation in the low‐recombining pericentromeric regions of wheat chromosomes spanning nearly 2/3 of the genome. The reduction of this deleterious genetic load is one of the possible strategies for crop improvement, and can likely be achieved by increasing the interstitial recombination rate. The identification of *trans*‐acting QTL in our study that can promote recombination in the pericentromeric regions provides the possibility for reducing deleterious genetic load in these low‐recombination regions of the wheat chromosomes.

## Experimental procedures

### Plant material

Twenty‐nine spring wheat accessions representing global and genetic diversity were chosen as parental lines (Table S1 and Figure 1a) to create a spring wheat nested association mapping (NAM) panel (Yu *et al*., 2008; Buckler *et al*., 2009). The common parent used to create the 28 families was photoperiod insensitive cultivar ‘Berkut’ that is included into the study because it represents one of the broadly adapted photoperiod insensitive cultivars developed by the international breeding program of CIMMYT. As most other NAM founders are non‐adapted wheat landraces, crossing with ‘Berkut’ allows for testing the phenotypic effects of landrace alleles in diverse environments. Recombinant inbred lines (RILs) for each cross were developed by self‐pollination for five generations using single seed descent. The F_6_ families were increased and evaluated for height and heading date to select the final set of 75 semi‐dwarf and early heading lines resulting in a population of 2100 RILs (Table S5).

A seed from each founder line and F_7_ NAM RIL was grown under greenhouse conditions. In total, 50 mg of plant tissue was collected from 2‐week‐old seedlings and DNA was extracted using the Qiagen DNeasy 96 Plant Kits (Qiagen, Venlo, Netherlands). Eluted DNA was quantified using Picogreen (Life Technologies, Carlsbad, CA, USA) and then normalized using the QIAgility robot (Qiagen) to obtain the concentration required for various sequencing and genotyping protocols.

### High‐density genotyping of the NAM founders

#### Sequence capture

The founder lines were analyzed using the Nimblegen exome capture assay as a part of the Wheat HapMap project (Jordan *et al*., 2015). Additional lines were sequenced using the same exome capture assay following the Wheat HapMap project protocols at the KSU Integrated Genomics Facility. The alignment and variant calling approaches applied and parameters applied were similar to those previously described in the Wheat HapMap project (Jordan *et al*., 2015), except for using the W7984 wheat reference genome assembly developed by Chapman *et al*. (2015) (Chapman *et al*., 2015). Similar to the Axiom dataset (see below), all heterozygous variants were treated as missing data. The filtered exome capture dataset retained 638 045 SNPs and 63 022 insertion/deletion polymorphisms (Tables S2 and S3) segregating in the NAM founders.

#### Axiom assay

The DNA of NAM parental lines was genotyped using an Axiom array resulting in genotype calls for 280 226 SNP loci. Filtering for polymorphic sites segregating across the 29 founders resulted in 149 685 SNPs (Table S4). Due to the low expected frequency of heterozygous sites in the F_7_ RILs, all heterozygous genotype calls were re‐called as missing data points. The Axiom array SNPs have been mapped to the W7984 assembly (Chapman *et al*., 2015). A set of 118 189 SNPs with no missing data was used to estimate the pairwise distances between the 29 NAM founders and construct the neighbor‐joining tree (Figure 1a) utilizing algorithms implemented in the R package *ape*. The pairwise distance corresponded to the proportion of SNPs different in each comparison.

The IBD regions between Berkut and founder lines was estimated using Beagle software (v. 4.1) (Browning and Browning, 2013) with the following parameters: window = 5000, overlap = 500, ibdlod = 3. The number of COs in the IBD regions in each population was compared with the number of COs in the regions showing no evidence of IBD using non‐parametric Mann–Whitney *U‐*test.

### Genotyping the NAM RILs and founders

#### Genotyping by sequencing

The libraries for sequencing were generated using the *Pst*I/*Mse*I combination of enzymes, as previously described (Saintenac *et al*., 2013), with the small modification, which includes performing size selection (270–300 bp) using the Pippin Prep system (Sage Scientific, Beverly, MA, USA) to further reduce the library complexity and generate higher read coverage for accurate polymorphism calling. Twenty‐six lanes of HiSeq 2500 sequencing were performed, followed by quality filtering and barcode separation (Table S5). To exclude the possibility that the structural divergence of the NAM founders from the reference genome will result in missing genotyping data due to unmappable reads, two pipelines capable of performing reference‐free variant detection were used. The pipelines used are our in‐house custom pipeline described in Saintenac *et al*. (2013) and the TASSEL UNEAK pipeline (Lu *et al*., 2013). The custom pipeline was also used to generate the presence–absence variation (PAV) calls, as described (Saintenac *et al*., 2013). The UNEAK pipeline was used with the default settings for bi‐parental mapping populations, and kept all polymorphisms that had no more than 90% data missing for the population. In summary, for each population, three sets of variant calls were derived: SNP calls from the custom pipeline, SNP calls from the UNEAK pipeline, and the presence–absence variation (PAV) calls from the custom pipeline. The three datasets were filtered further to ensure that both SNPs and PAVs segregate among the NAM parents and contain less than 20% missing data. The obtained variant calls from each dataset were combined with the SNP calls generated using the 90K iSelect assay (see below) to create family‐specific genetic maps. The variant calls with more than 20% missing data were combined with the PAV dataset and used for mapping into recombination bins of the newly created genetic maps following the previously described procedure (Saintenac *et al*., 2013).

Cross‐compared tag sequences across all populations using the BLAT program (Kent, 2002) were used to reduce the redundancy in the final GBS dataset included into the genetic maps. Tags that showed 100% identity or had only one mismatch that corresponded to the SNP call in the dataset were clustered together. This procedure lead to identifying 495 758 unique tag‐associated variants (164 668 SNPs and 331 090 PAV) that segregate in at least one bi‐parental mapping population (Tables S6 and S7).

#### 90K iSelect genotyping

In total, 500 ng of purified DNA of each founder and all NAM RILs was used for 90K iSelect genotyping (Wang *et al*., 2014) at the USDA Genotyping laboratory (Fargo). Raw image files were loaded into GenomeStudio Polyploid Clustering v1.0 (Wang *et al*., 2014) to create a project for each NAM family. Clustering followed the same three‐phase protocol as described in Wang *et al*. (2014) (Wang *et al*., 2014). The cutoff for allele frequency in clusters was ≥0.35 and ≤0.65, followed by manual curation of markers with multiple clusters. All markers that had exactly two clusters, a genotype call rate of ≥90%, and fell within the desired allele frequency range were retained. Further filtering was performed using a custom Perl script that eliminated markers in which the parents fell within the same cluster, or in which the mean of one cluster's Theta value (measure of angle of deviation from pure fluorescence signal) was less than two standard deviations away from the other cluster's Theta value. The final set of 57 657 high‐quality polymorphic SNPs that segregated in at least one bi‐parental mapping population (Table S8) was used for constructing family‐specific genetic maps.

Bi‐allelic variation of iSelect SNP markers across all populations was compared by a custom R script that used the boundaries of the genotype clusters (defined by mean and two standard deviations of Theta and R values of each cluster) in the bi‐parental mapping populations to assess the correctness of cluster assignments across all families. This procedure was utilized to ensure that all retained markers have the common parent's allele assigned to the same cluster position in all families, and resulted in 31 657 high‐quality bi‐allelic 90K iSelect markers (Table S9).

#### Cross‐comparison of markers across all populations

As GBS variant calling was performed on a per‐family basis, in total detecting 495 758 unique variants, SNP and PAV variant calling was repeated using the entire dataset by combining reads from all RILs. For this purpose, first, all GBS tags were clustered using CD‐Hit program at 97% identity generating 344 760 clusters. One tag from each cluster was used as a reference to map all raw reads from all individuals using Bowtie 2 v2.2.6 (Langmead and Salzberg, 2012) with program settings *fast* and *local*. Unified Genotyper from GATK v3.2‐2 (McKenna *et al*., 2010) was used to call SNPs across all 2100 NAM RILs and founders. Resulting variant calls were retained only if they matched the calls generated using the custom or UNEAK variant calling pipelines. Samtools v1.0 (Li *et al*., 2009) was used to count reads that aligned to sequence tags that corresponded to PAV. The present call was assigned if at least one read was mapped to a tag. The PAV were considered validated if the present‐absent calls matched the previous calls from the custom pipeline in the original mapping population that detected the PAV. Further, all SNP and PAV calls that matched between the previously generated calls made on a per‐family basis and the calls generated in the combined dataset, were tested for a 1:1 segregation ratio in individual families. Overall, 71 312 SNPs and 136 071 PAV were validated across the 2100 individuals (Table S7). Including bi‐allelic 90K SNP dataset, 235 599 markers (frequency above 1%) were identified and called across 2100 NAM RILs.

### Genetic maps

The UltraDense Mapping Software, Multipoint v3.3 ULD (Ronin *et al*., 2017) was used to group and order the genotype data in each population. In the first step, the Multipoint algorithm detects redundant markers with identical genotyping information across the RILs. Next, a single delegate marker for each redundant group was selected to have the least amount of missing data for the family and used for map construction applying the recombination frequency of 0.15 as a threshold for grouping into linkage groups. The linkage groups were assigned to chromosomes using the previously ordered 90K SNP markers from the consensus wheat genetic map (Wang *et al*., 2014). The developed genetic maps were used for QTL mapping within each family (Table S11).

All high‐quality redundant markers excluded from the map construction process due to redundancy were assigned the same chromosome and genetic position of the delegate marker that represented them. To add lower quality GBS markers with a higher proportion of missing data to the map, a custom Perl script was written to match the delegate markers based on 100% similarity of the genotype segregation patterns for the progeny present in the lower quality data. All matched SNPs were assigned the same chromosome and genetic position of the delegate markers. PAV tags were matched using the same custom Perl script only using the presence genotype calls and treating all absent genotypes as missing data. At least 15 PAV tags should have the present call and show 100% similarity to delegate marker tags to anchor a PAV to the genetic map. All matched PAV received the chromosome and genetic position of the delegate markers (Table S11).

### Mapping markers to reference genome scaffolds

All sequence tags and 90K SNP markers were mapped to the W7984 wheat genome scaffolds (Chapman *et al*., 2015) using the BLAT program (Kent, 2002). The W7984 reference genetic map is the short‐read sequence assembly genetically anchored to the Synthetic‐Opata M85 DH population. Markers were assigned the genetic position on the W7984 map if the best BLAT hit was on the same chromosome based on the NAM genetic map and had ≥95% similarity for ≥90% the length of sequence. Utilizing this approach, 63% of all markers were mapped to the reference genome. To obtain the locations of annotated genes on the W7984 map, previously annotated contigs of the wheat cultivar Chinese Spring (International Wheat Genome Sequencing Consortium, 2014) were mapped to PopSeq scaffolds (Chapman *et al*., 2015).

### Recombination events in the NAM population's RILs

A Perl script was written to count the total number of recombination events per chromosome for each individual in each family. Recombination events were counted as a change in the parental genotype phase ordered along the chromosome. The total number of CO events per RIL calculated from this script was used as the phenotype for subsequent QTL mapping strategies. We compared the total number of COs using both a single‐ and double‐marker parental genotype phase changes to define a CO event. We found the population average using single‐marker phase switch was 51.9 + 0.2 COs, and using two consecutive markers was 48.1 + 0.2 COs. The difference of 3.8 COs per RIL on average, represents two true double CO events, or two single mis‐genotyped or misplaced markers in the family genetic maps. We are unable to discern if or in which these occurrences happen within our dataset, hence we chose the conservative approach to define a CO as a change in parental genotype phase for two consecutive markers. Moreover, the selection of this CO estimation method is corroborated by the consistency of CO counts in our study with those estimated by immunolocalization of the MLH1 protein (48 + 4) (Martín *et al*., 2014). By reducing the probability of mis‐genotyped (error in genotype calling or alignment) or misplaced markers (error in map construction), potentially affecting our count of recombination events, we decrease false‐negative rate, but possibly miss few false positives, which could result from a true double crossover. By noting which markers are associated with the change of allele phase (CO event), it is possible to determine the incidence of crossovers at certain map positions based on the marker position on the reference genome (Table S13).

### Correlation of LD in founders with the meiotic recombination rate

Pairwise LD between all SNPs within the same recombination bin was estimated using the R package ‘genetics’. Linkage disequilibrium estimates for all pairwise combinations within recombination bins were averaged to get R^2^ value for each bin and was used to correlate with total recombination breakpoints within the same recombination bins using Pearson's correlation.

### Prediction of deleterious SNP alleles

Utilizing the segregating SNPs detected by exome capture among the founders, we identified potentially deleterious SNP alleles using the SNPeffect program (De Baets *et al*., 2012). In total, 42 965 SNPs located within the annotated gene models in the MIPS 2.2 genome annotation were mapped to the recombination bins of the NAM population genetic map. SNPs were considered potentially deleterious if the effect was determined ‘high’ using the SnpEff annotation criteria. The highly recombining regions were defined as the genetic recombination bins in the 10% distal ends of the chromosome that contained over 75% of the recombination events (Figure 2b). We compared the proportion of predicted deleterious to non‐deleterious SNPs in the highly recombining regions to the proportion of predicted deleterious to non‐deleterious SNPs in rest of the genome using one‐sided Fisher's exact test, formally testing for a reduction in genetic load.

### Recombination rate QTL mapping

The number of crossovers calculated from the custom Perl script (see above recombination events in NAM RILs), which counts a CO as a change in parental allele phase for two consecutive markers on each RIL's genetic map, was summed across all 21 wheat chromosomes to give the TCO phenotype for QTL mapping, similar to previous studies on the genetic control of recombination in wheat (Esch *et al*., 2007). QTL mapping was performed using QTL Cartographer v. 2.5_011 (statgen.ncsu.edu/qtlcart) using Composite Interval Mapping (CIM). Individuals that contained very high numbers of missing genotypes (>100 missing genotypes, ~10% total number of markers) were excluded from QTL analysis, and those individual's phenotypes were considered missing for mapping purposes. QTL mapping was performed using Model 6, the standard model. The stepwise regression method for marker co‐variates was forward selection/backward elimination with *P‐*value <0.01 for marker inclusion and exclusion, and a window size of 10 cM. QTL mapping was performed for each family separately using the TCO phenotype (Table S14). Genome‐wide significance thresholds were determined by permutations of genotype and phenotype data for each family. Two threshold levels, 0.05 and 0.0018 were applied; the latter threshold is Bonferroni‐corrected *P*‐value of 0.05/28. Confidence intervals to determine boundaries for bi‐parental QTL mapping regions were defined using 2 LOD intervals (Table S18).

### Additivity within bi‐parental mapping family QTL

Additivity of alleles was assessed for the 15 families in which more than one TCO QTL was mapped, and the four families in which more than one *trans*‐effect pCO QTL(see below) were detected. Genotypes of the most significantly associated markers were extracted and used to group RILs within the family based on the segregation patterns of the recombination favoring alleles within all the significant QTLs. Regression analyses were performed between phenotypic values and the number of favorable alleles. The resulting *R* ^2^ values and *P*‐values are presented in Table S20. The significance threshold *P *<* *0.05 and a positive regression were used to confirm additivity.

### *Cis*‐ and *trans*‐acting QTL

QTL acting in *cis‐* or *trans‐*configuration were determined based on previously described procedures (Esch *et al*., 2007). For this purpose, the TCO phenotypes were recalculated for each RIL after excluding the recombination events for the significant chromosomes, for pCO and dCO, phenotypes were recalculated for each RIL after excluding the respective portion of the chromosome carrying the QTL. For all recalculated values of TCO, pCO, and dCO another round of QTL mapping with QTL Cartographer v2.5.011, using the same parameters as original QTL for model selection, window size, and *P*‐value was carried out. Five percent significance thresholds were determined by permutation for each family. If the original QTL was detected again without the phenotypic contribution from the chromosome or chromosomal region harboring that QTL, we considered the QTL a *trans*‐acting QTL; if the original QTL was no longer detected, the QTL was considered a *cis*‐acting QTL (Tables S21 and S22).

### QTL affecting distribution of recombination events along the telomere‐centromere axis

Recombination events were calculated separately for the pericentromeric and distal regions of the wheat chromosome arms (pCOs and dCOs, respectively). The approximate genetic location of the centromere was estimated as previously described (Akhunov *et al*., 2013) using the sequences of flow sorted chromosome arm contigs (International Wheat Genome Sequencing Consortium, 2014). Using the position of centromere, each chromosome arm was divided into the distal (1/3 of chromosome arm away from centromere) and pericentromeric (2/3 of chromosome arms adjacent to the centromere) regions. This division was based on the previous estimates of recombination rate distribution that showed that most of the recombination is concentrated in the distal 1/3 of the wheat chromosome arms (Akhunov *et al*., 2003). Recombination rate phenotypes were estimated separately for distal and pericentromeric regions based on W7984 coordinates and were used for QTL mapping with ICIM software v 4.1.0.0 with default parameters stepping 1.0 cM and *P*‐value of 0.001. Five percent significance thresholds were determined by 1000 permutations (Table S22), and confidence intervals were defined using 2 LOD intervals. Additivity of QTL effects were determined in the same manner as described for bi‐parental TCO QTL in families that possessed more than one *trans*‐acting pericentromeric QTL.

### Joint inclusive composite interval mapping (JICIM)

The JICIM analysis in the NAM population was performed using both TASSEL v.5.2.42 and the ICIM v 4.1.0.0 program taking into account the nested family effect, following the procedures described by Buckler *et al*. (2009) (Buckler *et al*., 2009). The stepwise regression was run after marker genotypes were imputed using Beagle version b4.r1274, and input into the TASSEL stepwise function. In total, 17 273 markers were able to be ordered utilizing reference sequence and contained no missing data. Significant markers were selected by *P*‐value into the model using *P*‐value <0.0001 determined by permutation. The variance explained for the trait is the *R* ^*2*^ value for the stepwise regression model for each trait. JICIM mapping was performed using ICIM mapping software v4.1.0.0 on ordered markers with no missing data with settings of step size of 0.1 cM with a *P*‐value threshold of 0.001. Intervals were considered significant if the LOD was greater than 15.5 for both TCO and dCO, and greater than 14.0 for pCO, which were determined by permutation. Additive effects of each JICIM QTL were estimated using the ICIM software.

### Candidate genes affecting recombination

Gene models from the genomic regions spanning recombination QTL identified using different QTL mapping methods were extracted by using the annotation of the Chinese Spring reference genome (International Wheat Genome Sequencing Consortium, 2014). The extracted genes were functionally annotated using BLAST2GO v.4.1 (Conesa and Götz, 2008). Genes that have been previously shown to have an effect on recombination, or with functional annotation that corresponds to crossover initiation or chromatin remodeling, involved in DNA repair, DSB formation, or cytokinesis, kinetochore, spindle, or microtubule formation, or function as a topoisomerase were considered putative candidate genes.

### Availability of data and material

The raw sequence data can be accessed from the NCBI Short‐Read Archive database (BioProject SUB2540330 and PRJNA381058). All datasets associated with the paper can be downloaded from the project website: http://wheatgenomics.plantpath.ksu.edu/nam/.The genotype calls for NAM RILs have been deposited to the T3 database (triticeaetoolbox.org).

### Materials requests

Materials requests should be addressed to Eduard Akhunov (eakhunov@ksu.edu).

## Conflict of interest

The authors declare that they have no conflict of interests.

## Authors’ contributions

K.W.J. performed genotyping, map construction, QTL mapping and participated in drafting the manuscript. S.W. and F.H. performed bioinformatics data analyses. S.C. performed array‐based SNP genotyping, variant calling, and helped to select founder lines. Y.L. performed sequence capture. E.P. generated and analyzed Axiom assay genotyping data. P.S. contributed to identifying meiotic candidate genes. J.S. contributed to constructing genetic maps. A.A. contributed the exome capture sequencing and analyses. N.K.B. created NAM populations. M.P. created NAM populations and participated in data analyses. K.G. created NAM populations. J.D. participated in data analyses. L.T. created NAM populations and contributed to genetic map construction. E.A. conceived the idea, coordinated the project, analyzed data and wrote the manuscript.

## Supporting information


**Figure S1.** Genetic diversity of founder lines.
**Figure S2.** Genetic diversity of NAM RILs.
**Figure S3.** Workflow of genotyping and QTL mapping experiments.
**Figure S4**. Distribution of QTL and recombination breakpoints for homoeologous chromosome group 1.
**Figure S5**. Distribution of QTL and recombination breakpoints for homoeologous chromosome group 2.
**Figure S6.** Distribution of QTL and recombination breakpoints for homoeologous chromosomes group 3.
**Figure S7**. Distribution of QTL and recombination breakpoints for homoeologous chromosomes group 4.
**Figure S8**. Distribution of QTL and recombination breakpoints for homoeologous chromosomes group 5.
**Figure S9**. Distribution of QTL and recombination breakpoints for homoeologous chromosomes group 6.
**Figure S10**. Distribution of QTL and recombination breakpoints for homoeologous chromosomes group 7.
**Figure S11.** Distribution of recombination rate (cM/Mb), genetic diversity, and deleterious allele density across the wheat chromosomes.
**Figure S12.** Correlation of genetic distance among the NAM founders and recombination rate.
**Figure S13.** The additive allelic effects for TCO QTL.
**Figure S14.** The additive allelic effects for *trans*‐acting pCO QTL detected in bi‐parental mapping families.
**Figure S15**. The frequency of additive alleles across families detected by joint mapping.
**Figure S16.** The frequency of the non‐Berkut alleles with positive effect on recombination.

**Table S1**. List of founder lines used to create spring wheat NAM population.
**Table S2.** Exome capture SNPs detected in the founder lines.
**Table S3.** Exome capture inDels detected in the founder lines.
**Table S4.** Axiom array genotype calling of the founder lines.
**Table S5**. The list of recombinant inbred lines (RILs) that comprise the NAM population.
**Table S6.** The list of unique SNP and PAV GBS tag names after clustering and redundancy removal.
**Table S7.** The genotype matrix of all segregating bi‐allelic GBS SNPs and PAVs in the NAM population.
**Table S8.** 90K iSelect array genotype calling in the founder lines.
**Table S9.** The genotype matrix of all segregating bi‐allelic 90K iSelect SNPs in the NAM population.
**Table S10.** The number of markers genotyped using different technologies segregating in each NAM family.
**Table S11.** Twenty‐eight family‐specific genetic maps.
**Table S12.** Summary of genetic map lengths per chromosome and number of markers mapped per chromosome.
**Table S13**. Meiotic recombination breakpoints mapped to the recombination bins.
**Table S14**. Recombination phenotypes used for QTL mapping.
**Table S15.** Family‐specific summary of recombination traits used for QTL mapping.
**Table S16.** GBS SNP and PAV variation counts per recombination bin on the reference genome genetic map.
**Table S17.** The present call for PAV sites detected in the NAM founders with respect to reference genome.
**Table S18**. QTL mapping results in the individual NAM families.
**Table S19.** Overlap of QTL regions mapped in the individual NAM families.
**Table S20**. Additivity of recombination QTL.
**Table S21**. Classification of QTL into *cis*‐ and *trans*‐acting loci.
**Table S22**. QTL controlling the distribution of distal and pericentromeric CO.
**Table S23**. Stepwise regression (SR) analysis results.
**Table S24.** JCIM analyses and effect size estimates for each family.
**Table S25**. Marker estimates for significant stepwise regression markers by family.
**Table S26**. Regions of interest detected in multiple scans and multiple components of recombination.
**Table S27**. Conserved recombination candidate genes in regions of interest.
**Table S28**. Candidate genes with more than one homoeolog in a candidate region.
