# Supplementary material for: The genetic architecture of genome‐wide recombination rate variation in allopolyploid wheat revealed by nested association mapping
Source: Plant J. 2018 Jul 19;95(6):1039–54. doi: 10.1111/tpj.14009 (PMC6174997; doi:10.1111/tpj.14009)
Supplement: Supplementary file 1 — Figure S1. Genetic diversity of founder lines. Figure S2. Genetic diversity of NAM RILs. Figure S3. Workflow of genotyping and QTL mapping experiments. Figure S4. Distribution of QTL and recombination breakpoints for homoeologous chromosome group 1. Figure S5. Distribution of QTL and recombination breakpoints for homoeologous chromosome group 2. Figure S6. Distribution of QTL and recombination breakpoints for homoeologous chromosomes group 3. Figure S7. Distribution of QTL and recombination breakpoints for homoeologous chromosomes group 4. Figure S8. Distribution of QTL and recombination breakpoints for homoeologous chromosomes group 5. Figure S9. Distribution of QTL and recombination breakpoints for homoeologous chromosomes group 6. Figure S10. Distribution of QTL and recombination breakpoints for homoeologous chromosomes group 7. Figure S11. Distribution of recombination rate (cM/Mb), genetic diversity, and deleterious allele density across the wheat chromosomes. Figure S12. Correlation of genetic distance among the NAM founders and recombination rate. Figure S13. The additive allelic effects for TCO QTL. Figure S14. The additive allelic effects for trans‐acting pCO QTL detected in bi‐parental mapping families. Figure S15. The frequency of additive alleles across families detected by joint mapping. Figure S16. The frequency of the non‐Berkut alleles with positive effect on recombination. [file TPJ-95-1039-s001.docx]

**The genetic architecture and effects of genome-wide recombination rate distribution**

**in allopolyploid wheat**

Katherine W. Jordan, Shichen Wang, Fei He, Shiaoman Chao, Yanni Lun, Etienne Paux, Pierre Sourdille, Jamie Sherman, Alina Akhunova, Nancy K. Blake, Michael O. Pumphrey, Karl Glover, Jorge Dubcovsky, Luther Talbert, Eduard D. Akhunov

**Corresponding author:** Eduard Akhunov**,** Department of Plant Pathology**,** Kansas State University**,** Manhattan, KS 66506**,** e-mail: [eakhunov@ksu.edu](mailto:eakhunov@ksu.edu)**,** phone: 785-532-1342

**Supplementary Figures**

**Figure S1.** Genetic diversity of founder lines. **a**. Proportion of SNPs and Indels detected in the A (blue), B (orange), and D genomes (gray) using the whole exome capture (WEC) assay and Axiom genotyping array. **b**. The minor allele frequency of variants detected using wheat exome capture assay and Axiom genotyping array.

**Figure S2.** Genetic diversity in NAM RILs. **a**. Proportion of polymorphic markers detected for each family using different genotyping technologies and SNP calling pipelines. Variant calling in the GBS dataset was performed using two reference-free genotype calling algorithms: UNEAK and in-house custom GBS data processing pipeline described in Saintenac et al. (2013). The latter pipeline was used to call both SNPs and PAVs. HQ GBS and LC GBS correspond to SNPs with more than 80% (high quality) and 20% - 80% (low quality) data present, respectively. In addition to GBS, each RIL was genotyped using the 90K iSelect wheat array. **b**. The frequency of non-Berkut alleles in the NAM population. SNP and PAV genotyping was performed using different genotyping approaches as described above.

**Figure S3.** Workflow of genotyping and QTL mapping experiments.

**Figure S4**. Chromosomal distribution of mapped QTL and recombination breakpoints. Summary of QTL mapping results in 28 NAM families for the group 1 of homoeologous chromosomes. Each family is represented on the y-axis with horizontal dashed lines, starting with family 1 to 28. Red, blue, and yellow bars represent QTL regions for TCO, pCO, and dCO, respectively, detected in bi-parental mapping populations. Green squares represent significant SR and JCIM regions. Lower panel depicts the relationship between LD and the frequency of COs in the population across each chromosome.

**Figure S5**. Chromosomal distribution of mapped QTL and recombination breakpoints. Summary of QTL mapping results in 28 NAM families for the group 2 of homoeologous chromosomes. Each family is represented on the y-axis with horizontal dashed lines, starting with family 1 to 28. Red, blue, and yellow bars represent QTL regions for TCO, pCO, and dCO, respectively, detected in bi-parental mapping populations. Green squares represent significant SR and JCIM regions. Lower panel depicts the relationship between LD and the frequency of COs in the population across each chromosome.

**Figure S6.** Chromosomal distribution of mapped QTL and recombination breakpoints. Summary of QTL mapping results in 28 NAM families for the group 3 of homoeologous chromosomes. Each family is represented on the y-axis with horizontal dashed lines, starting with family 1 to 28. Red, blue, and yellow bars represent QTL regions for TCO, pCO, and dCO, respectively, detected in bi-parental mapping populations. Green squares represent significant JCIM regions. Lower panel depicts the relationship between LD and the frequency of COs in the population across each chromosome.

**Figure S7**. Chromosomal distribution of mapped QTL and recombination breakpoints. Summary of QTL mapping results in 28 NAM families for the group 4 of homoeologous chromosomes. Each family is represented on the y-axis with horizontal dashed lines, starting with family 1 to 28. Red, blue, and yellow bars represent QTL regions for TCO, pCO, and dCO, respectively, detected in bi-parental mapping populations. Green squares represent significant SR and JCIM regions. Lower panel depicts the relationship between LD and the frequency of COs in the population across each chromosome.

**Figure S8**. Chromosomal distribution of mapped QTL and recombination breakpoints. Summary of QTL mapping results in 28 NAM families for the group 5 of homoeologous chromosomes. Each family is represented on the y-axis with horizontal dashed lines, starting with family 1 to 28. Red, blue, and yellow bars represent QTL regions for TCO, pCO, and dCO, respectively, detected in bi-parental mapping populations. Green squares represent significant SR and JCIM regions. Lower panel depicts the relationship between LD and the frequency of COs in the population across each chromosome.

**Figure S9**. Chromosomal distribution of mapped QTL and recombination breakpoints. Summary of QTL mapping results in 28 NAM families for the group 6 of homoeologous chromosomes. Each family is represented on the y-axis with horizontal dashed lines, starting with family 1 to 28. Red, blue, and yellow bars represent QTL regions for TCO, pCO, and dCO, respectively, detected in bi-parental mapping populations. Green squares represent significant SR and JCIM regions. Lower panel depicts the relationship between LD and the frequency of recombination breakpoints in the population across each chromosome.

**Figure S10**. Chromosomal distribution of mapped QTL and recombination breakpoints. Summary of QTL mapping results in 28 NAM families for the group 7 of homoeologous chromosomes. Each family is represented on the y-axis with horizontal dashed lines, starting with family 1 to 28. Red, blue, and yellow bars represent QTL regions for TCO, pCO, and dCO, respectively, detected in bi-parental mapping populations. Green squares represent significant SR and JCIM. Lower panel depicts the relationship between LD and the frequency of COs in the population across each chromosome.

**Figure S11.** Distribution of recombination rate (cM/Mb), genetic diversity, and deleterious allele density across the wheat chromosomes. Deleterious allele load is the represented as the ratio of number of potentially deleterious SNPs to the total number of coding region SNPs for each genetic bin, and are scaled from 0 to 1 on the right y-axis, and depicted as black line. Recombination rates cM/Mb are increased on distal ends of the chromosomes, and scale is shown on left y-axis. Physical position was estimated for each marker located on bi-parental genetic map. Genetic and physical distance was calculated between adjacent markers on genetic maps, and given in cM/Mb units. Sliding window analysis was used to estimate the average recombination rate for 2Mb windows stepping every 1Mb across all NAM families. Scaled physical position was plotted on x-axis as the starting position of the window against the average recombination rate for the window. Recombination rate is depicted in blue, and genetic diversity is depicted in red. **a**. Homeologous chromosome group 1, **b**. Homeologous chromosome group 2, **c**. Homeologous chromosome group 3, **d**. Homeologous chromosome group 4, **e**. Homeologous chromosome group 5, **f**. Homeologous chromosome 6.

**Figure S12.**  Relatively weak correlation was found between the genetic distance among the founders estimated using SNP (a) and PAV (b) polymorphisms and the genome-wide recombination rate.

**Figure S13.** The additive allelic effects for recombination QTL. The number of recombination increasing alleles for TCO QTL correlate positively with the recombination rate for the two families with multiple detected QTL.

**Figure S14.** The additive allelic effects for *trans*-acting pCO QTL detected in bi-parental mapping families. Families with more than one pCO QTL were detected and grouped based on number of recombination favoring allele from the QTL analysis. The number of recombination favoring alleles for pCO QTL (blue) correlate positively with the recombination rate, while there is not a positive or significant correlation with the number of distal COs (red).

**Figure S15**. The frequency of additive alleles across families detected by joint mapping with different effect sizes for the TCO, dCO, and pCO QTL respectively. Positive alleles represent recombination favoring alleles from the common parent, while the negative alleles represent recombination favoring alleles from the other parent. The majority of QTL alleles had small effects across all traits.

**Figure S16.** The frequency of the non-Berkut alleles with positive effect on recombination. The recombination rate variation in wheat population is mostly controlled by rare QTL.
