# Supplementary material for: The genetic architecture of genome‐wide recombination rate variation in allopolyploid wheat revealed by nested association mapping
Source: Plant J. 2018 Jul 19;95(6):1039–54. doi: 10.1111/tpj.14009 (PMC6174997; doi:10.1111/tpj.14009)
Supplement: Supplementary file 2 — Table S1. List of founder lines used to create spring wheat NAM population. Table S2. Exome capture SNPs detected in the founder lines. Table S3. Exome capture inDels detected in the founder lines. Table S4. Axiom array genotype calling of the founder lines. Table S5. The list of recombinant inbred lines (RILs) that comprise the NAM population. Table S6. The list of unique SNP and PAV GBS tag names after clustering and redundancy removal. Table S7. The genotype matrix of all segregating bi‐allelic GBS SNPs and PAVs in the NAM population. Table S8. 90K iSelect array genotype calling in the founder lines. Table S9. The genotype matrix of all segregating bi‐allelic 90K iSelect SNPs in the NAM population. Table S10. The number of markers genotyped using different technologies segregating in each NAM family. Table S11. Twenty‐eight family‐specific genetic maps. Table S12. Summary of genetic map lengths per chromosome and number of markers mapped per chromosome. Table S13. Meiotic recombination breakpoints mapped to the recombination bins. Table S14. Recombination phenotypes used for QTL mapping. Table S15. Family‐specific summary of recombination traits used for QTL mapping. Table S16. GBS SNP and PAV variation counts per recombination bin on the reference genome genetic map. Table S17. The present call for PAV sites detected in the NAM founders with respect to reference genome. Table S18. QTL mapping results in the individual NAM families. Table S19. Overlap of QTL regions mapped in the individual NAM families. Table S20. Additivity of recombination QTL. Table S21. Classification of QTL into cis‐ and trans‐acting loci. Table S22. QTL controlling the distribution of distal and pericentromeric CO. Table S23. Stepwise regression (SR) analysis results. Table S24. JCIM analyses and effect size estimates for each family. Table S25. Marker estimates for significant stepwise regression markers by family. Table S26. Regions of interest detected in multiple scans and multiple com [file TPJ-95-1039-s002.docx]

**Supplementary Tables**

**(large files are not included in this document and may be accessed separately or downloaded separately from http://wheatgenomics.plantpath.ksu.edu/nam/)**

**Table S1**. Founder lines used to create spring wheat NAM population. The cultivar Berkut was used as the common parent and crossed to each of the other founders creating 28 NAM families.

| **Founder** | **NAM family** | **Origin** | **Status** |
| --- | --- | --- | --- |
| Berkut | all | Mexico | Cultivar |
| Dharwar Dry | NAM1 | India | Cultivar |
| PI 572692 | NAM2 | Georgia | Landrace |
| PI 283147 | NAM3 | Jordan | Landrace |
| PI 366716 | NAM4 | Afghanistan | Landrace |
| PI 382150 | NAM5 | Japan | Landrace |
| PI 470817 | NAM6 | Algeria | Landrace |
| PI 565213 | NAM7 | Bolivia | Landrace |
| PBW343 | NAM8 | India | Cultivar |
| Vida | NAM9 | USA | Cultivar |
| PI 9791 | NAM10 | Uzbekistan | Landrace |
| CI 4175 | NAM11 | Philippines | Landrace |
| CI 11223 | NAM12 | Croatia | Landrace |
| PI 262611 | NAM13 | Turkmenistan | Landrace |
| PI 70613 | NAM14 | China | Landrace |
| CI 7635 | NAM15 | Russia | Landrace |
| PI 8813 | NAM16 | Iraq | Landrace |
| PI 82469 | NAM17 | North Korea | Landrace |
| CI 15144 | NAM18 | Saudi Arabia | Landrace |
| PI 43355 | NAM19 | Uruguay | Landrace |
| PI 94567 | NAM20 | Israel | Landrace |
| PI 192001 | NAM23 | Angola | Landrace |
| PI 192147 | NAM24 | Ethiopia | Landrace |
| CI 15134 | NAM25 | Pakistan | Landrace |
| PI 185715 | NAM26 | Portugal | Landrace |
| PI 192569 | NAM27 | Sweden | Landrace |
| PI 210945 | NAM28 | Cyprus | Landrace |
| PI 220431 | NAM29 | Egypt | Landrace |
| PI 278297 | NAM30 | Greece | Landrace |

**Table S2.** Genotype matrix of exome capture SNPs detected in the founder lines (separate tab delimited file, download full dataset from http://wheatgenomics.plantpath.ksu.edu/nam/).

**Table S3.** Genotype matrix of exome capture indels detected in the founder lines (separate tab delimited file, download full dataset from http://wheatgenomics.plantpath.ksu.edu/nam/).

**Table S4.** Genotype matrix of Axiom array calls of the founder lines (separate tab delimited file, download full dataset from http://wheatgenomics.plantpath.ksu.edu/nam/).

**Table S5**. The list of recombinant inbred lines (RILs) that comprise the NAM population with cross information (separate Excel file, download from http://wheatgenomics.plantpath.ksu.edu/nam/).

**Table S6.** The list of unique SNP and PAV GBS tag names after clustering and redundancy removal (separate tab delimited file, download full dataset from http://wheatgenomics.plantpath.ksu.edu/nam/).

**Table S7.** The genotype matrix of all segregating biallelic GBS SNPs and PAVs in the NAM population (separate tab delimited file, download full dataset from http://wheatgenomics.plantpath.ksu.edu/nam/).

**Table S8.** 90K iSelect array genotype calling of the founder lines (separate tab delimited file, download full dataset from http://wheatgenomics.plantpath.ksu.edu/nam/).

**Table S9.** The genotype matrix of all segregating biallelic 90K iSelect SNPs in the NAM population (separate tab delimited file, download full dataset from http://wheatgenomics.plantpath.ksu.edu/nam/).

**Table S10.** The number of markers genotyped using different technologies segregating in each NAM family.

| **Family** | **Parent** | **90K** | **SNP** | **PAV** | **Total Mapped** |
| --- | --- | --- | --- | --- | --- |
| NAM1 | Dharwar Dry | 12234 | 13564 | 24064 | 49862 |
| NAM2 | PI572692 | 11273 | 10892 | 36317 | 58482 |
| NAM3 | PI283147 | 10451 | 9887 | 31716 | 52054 |
| NAM4 | PI366716 | 10428 | 18718 | 32344 | 61490 |
| NAM5 | PI382150 | 11367 | 22265 | 32523 | 66155 |
| NAM6 | PI470817 | 9661 | 14736 | 37824 | 62221 |
| NAM7 | PI565213 | 7427 | 9882 | 14325 | 31634 |
| NAM8 | PBW343 | 10051 | 10593 | 27790 | 48434 |
| NAM9 | Vida | 12784 | 10156 | 33355 | 56295 |
| NAM10 | PI9791 | 12614 | 10344 | 18969 | 41927 |
| NAM11 | Cltr4175 | 11259 | 13133 | 37437 | 61829 |
| NAM12 | Cltr11223 | 11055 | 10684 | 35084 | 56823 |
| NAM13 | PI262611 | 11945 | 24318 | 31111 | 67374 |
| NAM14 | PI70613 | 9437 | 7846 | 22718 | 40001 |
| NAM15 | Cltr7635 | 10200 | 8491 | 28905 | 47596 |
| NAM16 | PI8813 | 8082 | 6031 | 23090 | 37203 |
| NAM17 | PI82469 | 11170 | 14190 | 25171 | 50531 |
| NAM18 | Cltr15144 | 7239 | 5035 | 14712 | 26986 |
| NAM19 | PI43355 | 12087 | 11822 | 32679 | 56588 |
| NAM20 | PI94567 | 5996 | 5473 | 16887 | 28356 |
| NAM23 | PI192001 | 9569 | 8216 | 33674 | 51459 |
| NAM24 | PI192147 | 9459 | 12733 | 42650 | 64842 |
| NAM25 | Cltr15134 | 7271 | 6017 | 29242 | 42530 |
| NAM26 | PI185715 | 10947 | 12104 | 31754 | 54805 |
| NAM27 | PI192569 | 10155 | 9244 | 26578 | 45977 |
| NAM28 | PI210945 | 11080 | 8774 | 29936 | 49790 |
| NAM29 | PI220431 | 10716 | 9823 | 25612 | 46151 |
| NAM30 | PI278297 | 10075 | 9771 | 26774 | 46620 |
| Average |  | 10215 | 11241 | 28687 | 50143 |

**Table S11.** Twenty-eight family-specific genetic maps constructed using *Multipoint* software (separate tab delimited file, download all family maps from http://wheatgenomics.plantpath.ksu.edu/nam/).

**Table S12.** Summary of genetic map lengths per chromosome and number of markers mapped per chromosome for each of the NAM families (separate Excel file, download from http://wheatgenomics.plantpath.ksu.edu/nam/).

**Table S13**. Meiotic recombination breakpoints mapped to the W7984 reference genome assembly recombination bins (separate Excel file, download full dataset from http://wheatgenomics.plantpath.ksu.edu/nam/).

**Table S14**. Recombination phenotypes used for QTL mapping (separate Excel file, download from http://wheatgenomics.plantpath.ksu.edu/nam/).

**Table S15.** Family-specific summary of TCO phenotype used for QTL mapping.

| **Population** | **Trait Name** | **Sample Size** | **Mean** | **Variance** | **StdError** | **Minimum** | **Maximum** | **Range** |
| --- | --- | --- | --- | --- | --- | --- | --- | --- |
| **NAM1** | TCO | 75 | 43.75 | 62.16 | 7.88 | 30 | 62 | 32 |
| **NAM2** | TCO | 72 | 52.21 | 63.15 | 7.95 | 39 | 76 | 37 |
| **NAM3** | TCO | 74 | 48.04 | 50.78 | 7.13 | 32 | 67 | 35 |
| **NAM4** | TCO | 60 | 49.67 | 68.63 | 8.28 | 35 | 74 | 39 |
| **NAM5** | TCO | 70 | 47.27 | 40.08 | 6.33 | 36 | 66 | 30 |
| **NAM6** | TCO | 73 | 51.62 | 50.71 | 7.12 | 34 | 75 | 41 |
| **NAM7** | TCO | 70 | 48.57 | 43.12 | 6.57 | 35 | 66 | 31 |
| **NAM8** | TCO | 71 | 37.48 | 43.20 | 6.57 | 21 | 52 | 31 |
| **NAM9** | TCO | 73 | 48.19 | 45.05 | 6.71 | 31 | 64 | 33 |
| **NAM10** | TCO | 72 | 48.06 | 53.12 | 7.29 | 34 | 70 | 36 |
| **NAM11** | TCO | 74 | 50.86 | 69.60 | 8.34 | 33 | 75 | 42 |
| **NAM12** | TCO | 68 | 47.38 | 53.55 | 7.32 | 33 | 70 | 37 |
| **NAM13** | TCO | 72 | 47.14 | 49.25 | 7.02 | 26 | 65 | 39 |
| **NAM14** | TCO | 68 | 47.38 | 65.52 | 8.09 | 28 | 79 | 51 |
| **NAM15** | TCO | 71 | 44.52 | 37.80 | 6.15 | 29 | 59 | 30 |
| **NAM16** | TCO | 71 | 49.56 | 43.42 | 6.59 | 36 | 67 | 31 |
| **NAM17** | TCO | 71 | 48.76 | 46.07 | 6.79 | 29 | 64 | 35 |
| **NAM18** | TCO | 74 | 47.86 | 33.71 | 5.81 | 35 | 62 | 27 |
| **NAM19** | TCO | 74 | 48.43 | 85.02 | 9.22 | 31 | 76 | 45 |
| **NAM20** | TCO | 72 | 45.38 | 36.10 | 6.01 | 30 | 59 | 29 |
| **NAM23** | TCO | 71 | 45.01 | 34.93 | 5.91 | 34 | 60 | 26 |
| **NAM24** | TCO | 70 | 55.67 | 58.92 | 7.68 | 38 | 74 | 36 |
| **NAM25** | TCO | 67 | 48.73 | 37.38 | 6.11 | 34 | 64 | 30 |
| **NAM26** | TCO | 64 | 48.30 | 38.31 | 6.19 | 32 | 61 | 29 |
| **NAM27** | TCO | 72 | 52.85 | 42.30 | 6.50 | 36 | 65 | 29 |
| **NAM28** | TCO | 70 | 46.91 | 56.25 | 7.50 | 29 | 69 | 40 |
| **NAM29** | TCO | 73 | 45.93 | 37.70 | 6.14 | 31 | 64 | 33 |
| **NAM30** | TCO | 71 | 46.90 | 58.95 | 7.68 | 29 | 69 | 40 |
|  |  |  |  |  |  |  |  |  |

**Table S16.** GBS SNP and PAV variation counts per recombination bin on the W7984 reference genome genetic map (separate Excel file, download from http://wheatgenomics.plantpath.ksu.edu/nam/).

**Table S17.** The present call for PAV sites detected in the NAM founders with respect to reference genome (separate Excel file, download from http://wheatgenomics.plantpath.ksu.edu/nam/).

**Table S18**. QTL mapping results in the individual NAM families. The map locations in individual mapping populations were converted to the coordinates of the W7984 wheat genome assembly.

| **Family** | **Chr** | **QTL pos** | **PeakMarker** | **LR** | **Significance (p)*** | **LOD** | **R2** | **Add†** | **Chr** | **Reference Position cM‡** |
| --- | --- | --- | --- | --- | --- | --- | --- | --- | --- | --- |
| NAM1 | 2A | 8.6-30.8 | 7 | 24.99 | 0.0018 | 5.4 | 18.5 | 3.56 | 2A | 58.902-92.51 |
|  | 7A | 74.9-82.4 | 38 | 16.41 | 0.05 | 3.6 | 12.9 | -2.89 | 7A | 40.05-43.46 |
| NAM2 | 1D | 143.0-155.8 | 24 | 21.6 | 0.05 | 4.7 | 17.2 | -3.35 | 1D | 35.26-54.588 |
| NAM4 | 7B | 156.2-161.3 | 81 | 41.09 | 0.0018 | 8.9 | 34.8 | -9.12 | 7B | 51.193-118.55 |
|  | 7B | 169.1-179.2 | 97 | 36.5 | 0.0018 | 7.9 | 37.2 | 9.21 | 7B | 111.73-118.55 |
| NAM5 | 6B | 53.1-55.0 | 15 | 14.08 | 0.05 | 3.1 | 12.7 | 2.41 | 6B | 35.322-47.831 |
| NAM6 | 7B | 135.9-146.1 | 52 | 16.64 | 0.05 | 3.6 | 16.4 | 3.11 | 7B | 95.79-101.484 |
| NAM7 | 4A | 152.9-156.5 | 41 | 13.97 | 0.05 | 3.0 | 10.2 | -3.42 | 4A | 111.17-131.64 |
|  | 7B | 111.1-119.0 | 42 | 17.2 | 0.0018 | 3.7 | 12.8 | 4.12 | 7B | 94.66-101.484 |
| NAM8 | 1A | 78.4-94.4 | 38 | 20.13 | 0.05 | 4.4 | 17.3 | 3.08 | 1A | 110.827-117.88 |
|  | 5A | 79.3-89.3 | 31 | 17.68 | 0.05 | 3.8 | 15.5 | -2.64 | 5A | 9.09-13.64 |
| NAM9 | 7B | 73.5-77.6 | 30 | 15.3 | 0.05 | 3.3 | 12.9 | -2.61 | 7B | 51.193-59.156 |
| NAM12 | 2B | 16.1-22.4 | 10 | 14.89 | 0.05 | 3.2 | 12.9 | -2.66 | 2B | 15.931-18.204 |
|  | 5D | 75.7-89.1 | 16 | 36 | 0.0018 | 7.8 | 31.8 | -4.23 | 5D | 159.32-168.43 |
| NAM14 | 3A | 0-12.7 | 4 | 18.8 | 0.05 | 4.1 | 20.2 | -3.74 | 3A | 9.11-17.08 |
|  | 6B | 51.1-57.0 | 19 | 14.76 | 0.05 | 3.2 | 14.1 | 3.29 | 6B | 46.694-47.83 |
| NAM15 | 1B | 9.7-17.2 | 9 | 17.6 | 0.05 | 3.8 | 14.5 | 2.45 | 1B | 7.962-44.438 |
|  | 7B | 0-15.5 | 2 | 33.6 | 0.0018 | 7.3 | 29.9 | -3.38 | 7B | 7.97-45.51 |
| NAM16 | 4A | 55.2-59.3 | 20 | 17.7 | 0.05 | 3.8 | 15.3 | 2.67 | 4A | 61.015-74.1275 |
|  | 5A | 149.0-175.4 | 51 | 32.9 | 0.0018 | 7.2 | 30.1 | -3.73 | 5A | 47.99-86.61 |
| NAM17 | 2B | 103.8-109.6 | 61 | 17.2 | 0.05 | 3.7 | 18 | -3.02 | 2B | 59.184-96.718 |
| NAM18 | 5B | 53.2-71.5 | 23 | 30.54 | 0.0018 | 6.6 | 22 | 2.78 | 5B | 39.90-80.923 |
|  | 6A | 19.3-93.4 | 9 | 27.5 | 0.05 | 6.0 | 36.7 | -3.61 | 6A | 46.79-61.582 |
| NAM19 | 4A | 90.3-93.6 | 32 | 14.26 | 0.0018 | 3.1 | 10.8 | -3.22 | 4A | 103.282-111.18 |
|  | 4B | 13.5-31.5 | 7 | 21.22 | 0.05 | 4.6 | 15.7 | -3.86 | 4B | 19.662-61.749 |
| NAM20 | 4B | 128.1-130.1 | 38 | 15.75 | 0.05 | 3.4 | 13.7 | 2.35 | 4B | 93.03-99.28 |
|  | 7A | 0-9.8 | 8 | 18.78 | 0.05 | 4.1 | 16.5 | 2.55 | 7A | 0-32.019 |
| NAM23 | 1B | 126.9-137.4 | 65 | 17.23 | 0.05 | 3.7 | 14.6 | -2.3 | 1B | 91.06-100.17 |
| NAM24 | 2A | 183.2-190.2 | 58 | 13.94 | 0.05 | 3.0 | 13.5 | 2.86 | 2A | 58.66-92.517 |
| NAM25 | 6A | 143.7-148.1 | 57 | 17.26 | 0.05 | 3.8 | 14.1 | -2.39 | 6A | 88.019-98.254 |
|  | 7B | 45.9-61.8 | 16 | 19.3 | 0.05 | 4.2 | 15.5 | -2.61 | 7B | 50.62-64.839 |
| NAM26 | 7A | 0-3.3 | 3 | 18.31 | 0.05 | 4.0 | 15.9 | -2.5 | 7A | 0-32.019 |
|  | 7A | 92.8-103.4 | 27 | 22.31 | 0.0018 | 4.9 | 20.4 | -2.99 | 7A | 58.263-62.809 |
| NAM27 | 2D | 104.7-121.2 | 11 | 20.88 | 0.05 | 4.5 | 15.3 | 2.66 | 2D | 11.36- 20.69 |
|  | 5A | 51.3-77.3 | 8 | 14.01 | 0.05 | 3.0 | 34 | -3.85 | 5A | 91.39-93.66 |
|  | 6A | 164.2-186.0 | 46 | 31.2 | 0.0018 | 6.8 | 26.1 | 3.39 | 6A | 69.72-98.8225 |
| NAM30 | 2D | 0-0.7 | 1 | 14.56 | 0.05 | 3.2 | 9.6 | -2.42 | 2D | 54.33-56.609 |
|  | 4A | 111.0-126.3 | 52 | 30.5 | 0.0018 | 6.6 | 22.7 | 3.86 | 4A | 103.282-112.32 |
|  | 4B | 4.4-29.4 | 9 | 22.04 | 0.0018 | 4.8 | 15.9 | -3.15 | 4B | 6.843-39 |
|  | 6B | 4.5-8.1 | 6 | 15.36 | 0.05 | 3.3 | 10.1 | 2.49 | 6B | 9.097-43.284 |

* Significance threshold for QTL confidence; 0.05 is used as general measure of significance within family, 0.0018 is the Bonferroni significance for testing in 28 families

† Effect is positive; the common parent allele (Berkut) favors more recombination than other parent. Negative effect the Berkut allele shows less recombination than other parent

‡W7984 coordinates given by taking markers located within the bi-parental QTL region and matching corresponding boundaries on W7984 reference scaffolds

**Table S19.** Overlap of QTL regions mapped in the individual NAM families.

| **Trait** | **Families** | **Chr** | **W7984 cM†** | **Population** | **Effect‡** |
| --- | --- | --- | --- | --- | --- |
| TCO | 2 | 2A | 58.902-92.51 | NAM1 , NAM24 | +/+ |
| TCO | 2 | 4A | 103.282-111.18 | NAM19 , NAM30 | -/+ |
| TCO | 3 | 4A | 111.18 | NAM7, NAM19, NAM30 | -/-/+ |
| TCO | 2 | 4B | 19.662-39 | NAM19, NAM30 | -/- |
| TCO | 2 | 6A | 88.019-98.254 | NAM25, NAM27 | -/+ |
| TCO | 2 | 6B | 35.322-47.831 | NAM30, NAM5, NAM14 | +/+/+ |
| TCO | 2 | 7A | 0-32.019 | NAM20, NAM26 | +/- |
| TCO | 3 | 7B | 51.193-59.156 | NAM4, NAM9, NAM25 | +/+/+ |
| TCO | 3 | 7B | 95.79-109.46 | NAM4, NAM6, NAM7 | -/+/+ |
| pCO | 2 | 1B | 30.7-44.4 | NAM5, NAM13 | +/- |
| pCO | 2 | 1B | 48.9-63.7 | NAM13, NAM28 | +/+ |
| pCO | 2 | 2D | 66.8-73.6 | NAM10, NAM19 | -/- |
| pCO | 2 | 5B | 38.7-45.6 | NAM6, NAM23 | +/+ |
| pCO | 2 | 5B | 70.6-79.8 | NAM6, NAM29 | +/- |
| pCO | 2 | 6B | 27.4-35.3 | NAM5, NAM6 | +/+ |
| dCO | 2 | 3A | 17.079 | NAM1, NAM14 | +/- |
| dCO | 2 | 6B | 35-37 | NAM8, NAM25 | -/+ |
| dCO | 2 | 6B | 46-47.831 | NAM14/NAM25 | +/+ |

| † These represent the narrow range of overlap |
| --- |
| ‡ For positive effects the recombination favoring allele comes from the common parent Berkut, negative effects occur when the recombination favoring allele comes from the other parent |

**Table S20**. Additivity of recombination QTL.

| **Family** | **Trait** | **R^2†^** | **Corr** | **P-value** | **Number QTL ^‡^** | | **Phenotypic Range^§^** |
| --- | --- | --- | --- | --- | --- | --- | --- |
| NAM1 | TCO | 0.34 | 0.59 | 6.13E-08 | 2 | | 12.71 |
| NAM4 | TCO | 0.004 | -0.07 | 0.6209 | 2 | | -2.5 |
| NAM7 | TCO | 0.19 | 0.43 | 0.000187 | 2 | | 7.71 |
| NAM8 | TCO | 0.25 | 0.5 | 8.28E-06 | 2 | | 9.975 |
| NAM12 | TCO | 0.25 | 0.5 | 1.44E-05 | 2 | | 10.45 |
| NAM14 | TCO | 0.19 | 0.43 | 0.000344 | 2 | | 10.79 |
| NAM15 | TCO | 0.15 | 0.39 | 0.000812 | 2 | | 6.55 |
| NAM16 | TCO | 0.22 | 0.47 | 5.94E-05 | 2 | | 9.82 |
| NAM18 | TCO | 0.35 | 0.6 | 1.96E-08 | 2 | | 9.77 |
| NAM19 | TCO | 0.21 | 0.46 | 4.42E-05 | 2 | | 12.89 |
| NAM20 | TCO | 0.14 | 0.37 | 0.0014 | 2 | | 7.08 |
| NAM25 | TCO | 0.24 | 0.49 | 2.15E-05 | 2 | | 9.25 |
| NAM26 | TCO | 0.21 | 0.46 | 0.0001 | 2 | | 8.34 |
| NAM27 | TCO | 0.29 | 0.54 | 1.21E-06 | 3 | | 13.98 |
| NAM30 | TCO | 0.48 | 0.69 | 3.47E-11 | 4 | | 12.23 |
| NAM6 | pCO | 0.28 | 0.53 | 1.80E-06 | 2 | | 3.91 |
| NAM8 | pCO | 0.3 | 0.54 | 1.16E-06 | 2 | | 3.63 |
| NAM10 | pCO | 0.2 | 0.45 | 6.12E-05 | 3 | | 4.63 |
| NAM29 | pCO | 0.37 | 0.61 | 1.37E-08 | 3 | | 5.88 |
| † R2 value represents a purely additive model. | | | | | | |  |
| ^‡^ Number of QTL in this family for this trait | | | | | | |  |
| ^§^ Range in effect size from zero beneficial alleles to all beneficial alleles | | | | | | |  |

**Table S21**. Classification of TCO QTL into *cis*- and *trans*-acting loci.

| **Family** | **Chr** | **QTL pos** | **Marker** | **LOD** | **W7984 cM†** | **cis/trans** | **Chr** | **LR** | **Marker** |
| --- | --- | --- | --- | --- | --- | --- | --- | --- | --- |
| NAM1 | 2A | 8.6-30.8 | 7 | 5.43 | 58.902-92.51 | trans | 2A | 4.60 | 7 |
|  | 7A | 74.9-82.4 | 38 | 3.57 | 40.05-43.46 | trans | 7A | 4.17 | 38 |
| NAM2 | 1D | 143.0-155.8 | 24 | 4.70 | 35.26-54.588 | trans | 1D | 7.97 | 25 |
| NAM4 | 7B | 156.2-161.3 | 81 | 8.93 | 51.193-118.55 | cis | not detected |  |  |
|  | 7B | 169.1-179.2 | 97 | 7.93 | 111.73-118.55 | cis | not detected |  |  |
| NAM5 | 6B | 53.1-55.0 | 15 | 3.06 | 35.322-47.831 | cis | not detected |  |  |
| NAM6 | 7B | 135.9-146.1 | 52 | 3.62 | 95.79-101.484 | cis | not detected |  |  |
| NAM7 | 4A | 152.9-156.5 | 41 | 3.04 | 111.17-131.64 | trans | 4A | 3.50 | 41 |
|  | 7B | 111.1-119.0 | 42 | 3.74 | 94.66-101.484 | trans | 7B | 3.20 | 42 |
| NAM8 | 1A | 78.4-94.4 | 38 | 4.38 | 110.827-117.88 | trans | 1A | 4.48 | 38 |
|  | 5A | 79.3-89.3 | 31 | 3.84 | 9.09-13.64 | trans | 5A | 3.98 | 31 |
| NAM9 | 7B | 73.5-77.6 | 30 | 3.33 | 51.193-59.156 | trans | 7B | 3.24 | 60 |
| NAM12 | 2B | 16.1-22.4 | 10 | 3.24 | 15.931-18.204 | trans | 2B | 3.85 | 10 |
|  | 5D | 75.7-89.1 | 16 | 7.83 | 159.32-168.43 | trans | 5D | 7.14 | 16 |
| NAM14 | 3A | 0-12.7 | 4 | 4.09 | 9.11-17.08 | trans | 3A | 4.98 | 4 |
|  | 6B | 51.1-57.0 | 19 | 3.21 | 46.694-47.83 | trans | 6B | 4.70 | 21 |
| NAM15 | 1B | 9.7-17.2 | 9 | 3.83 | 7.962-44.438 | trans | 1B | 2.77 | 9 |
|  | 7B | 0-15.5 | 2 | 7.30 | 7.97-45.51 | trans | 7B | 5.49 | 3 |
| NAM16 | 4A | 55.2-59.3 | 20 | 3.85 | 61.015-74.1275 | trans | 4A | 4.28 | 20 |
|  | 5A | 149.0-175.4 | 51 | 7.15 | 47.99-86.61 | trans | 5A | 3.92 | 51 |
| NAM17 | 2B | 103.8-109.6 | 61 | 3.74 | 59.184-96.718 | cis | not detected |  |  |
| NAM18 | 5B | 53.2-71.5 | 23 | 6.64 | 39.90-80.923 | trans | 5B | 4.62 | 22 |
|  | 6A | 19.3-93.4 | 9 | 5.98 | 46.79-61.582 | trans | 6A | 7.43 | 9 |
| NAM19 | 4A | 90.3-93.6 | 32 | 3.10 | 103.282-111.18 | trans | 4A | 2.69 | 32 |
|  | 4B | 13.5-31.5 | 7 | 4.61 | 19.662-61.749 | cis | not detected |  |  |
| NAM20 | 4B | 128.1-130.1 | 38 | 3.42 | 93.03-99.28 | cis | not detected |  |  |
|  | 7A | 0-9.8 | 8 | 4.08 | 0-32.019 | cis | not detected |  |  |
| NAM23 | 1B | 126.9-137.4 | 65 | 3.75 | 91.06-100.17 | trans | 1B | 2.97 | 64 |
| NAM24 | 2A | 183.2-190.2 | 58 | 3.03 | 58.66-92.517 | trans | 2A | 4.87 | 58 |
| NAM25 | 6A | 143.7-148.1 | 57 | 3.75 | 88.019-98.254 | trans | 6A | 2.97 | 57 |
|  | 7B | 45.9-61.8 | 16 | 4.20 | 50.62-64.839 | cis | not detected |  |  |
| NAM26 | 7A | 0-3.3 | 3 | 3.98 | 0-32.019 | cis | not detected |  |  |
|  | 7A | 92.8-103.4 | 27 | 4.85 | 58.263-62.809 | trans | 7A | 4.53 | 29 |
| NAM27 | 2D | 104.7-121.2 | 11 | 4.54 | 11.36- 20.69 | trans | 2D | 3.05 | 11 |
|  | 5A | 51.3-77.3 | 8 | 3.05 | 91.39-93.66 | cis | not detected |  |  |
|  | 6A | 164.2-186.0 | 46 | 6.78 | 69.72-98.8225 | trans | 6A | 5.46 | 46 |
| NAM30 | 2D | 0-0.7 | 1 | 3.17 | 54.33-56.609 | trans | 2D | 3.67 | 1 |
|  | 4A | 111.0-126.3 | 52 | 6.63 | 103.282-112.32 | trans | 4A | 6.72 | 52 |
|  | 4B | 4.4-18.4 | 9 | 4.79 | 6.843-32.176 | trans | 4B | 5.94 | 9 |
|  | 6B | 4.5-8.1 | 6 | 3.34 | 9.097-43.284 | cis | not detected |  |  |

†W7984 coordinates given by taking markers that were on the bi-parental map and matching locations on W7984 reference genome assembly scaffolds.

**Table S22**. QTL controlling the distribution of distal and pericentromeric CO.

| **Family** | **Trait** | **LeftMarker** | | **RightMarker** | | **LOD** | **PVE** | **Add** | **cis/trans** | **Chr** | **W7984 Range†** |
| --- | --- | --- | --- | --- | --- | --- | --- | --- | --- | --- | --- |
| NAM1 | dCO | | IAAV6070 | | RAC875_c9523_328 | 4.8 | 23.9 | 2.16 | cis | 2A | 91.38-92.517 |
|  | dCO | | wsnp_Ku_c7060_12212702 | | wsnp_Ra_rep_c106523_90273922 | 3.1 | 13.3 | 1.64 | trans | 3A | 11.391-17.079 |
| NAM5 | pCO | | wsnp_Ku_c2620_4980121 | | wsnp_Ex_c14273_22230844 | 3.2 | 16.5 | -1.06 | cis | 1B | 25.027-44.438 |
|  | pCO | | RAC875_c19252_548 | | Tdurum_contig58912_605 | 4.2 | 21.7 | 1.23 | trans | 6B | 20.534-67.16 |
| NAM6 | pCO | | BS00021960_51 | | SpringWheatNAM_tag_1978 | 3.3 | 16.0 | 1.04 | trans | 5B | 38.769-109.35 |
|  | pCO | | BS00061749_51 | | BS00065500_51 | 3.4 | 16.1 | 1.04 | trans | 6B | 27.36-35.322 |
| NAM8 | pCO | | SpringWheatNAM_tag_41636 | | SpringWheatNAM_tag_100280 | 5.8 | 28.5 | -1.22 | trans | 6A | 30.838-31.4065 |
|  | pCO | | GENE-4796_65 | | SpringWheatNAM_tag_236640 | 3.4 | 15.5 | 0.90 | trans | 7D | 104.961-148.35 |
|  | dCO | | BobWhite_c35035_317 | | RAC875_c26177_632 | 3.0 | 59.1 | -2.71 | cis | 6B | 35.322-37.0275 |
| NAM10 | pCO | | Kukri_c5283_1035 | | Ex_c14755_1362 | 3.0 | 28.0 | -1.77 | trans | 2D | 13.642-85.027 |
|  | pCO | | Kukri_c869_442 | | Kukri_c22559_916 | 3.4 | 14.3 | -1.22 | cis | 4B | 58.338-60.612 |
|  | pCO | | Kukri_rep_c72957_431 | | RAC875_c52560_123 | 4.5 | 19.8 | 1.45 | trans | 7A | 21.855-28.677 |
| NAM11 | pCO | | D_contig35269_394 | | wsnp_Ra_c9738_16173810 | 2.8 | 15.1 | -1.00 | cis | 3A | 17.079-20.49 |
| NAM12 | dCO | | SpringWheatNAM_tag_42419 | | Excalibur_c6552_727 | 3.0 | 16.2 | -2.03 | trans | 5D | 159.327-165.014 |
| NAM13 | pCO | | BS00022902_51 | | Excalibur_c20072_758 | 4.5 | 23.5 | 1.47 | cis | 1B | 30.711-66.042 |
|  | pCO | | RAC875_c6805_1347 | | Ku_c12701_1273 | 4.2 | 20.3 | -1.40 | trans | 7A | 36.64-51.442 |
| NAM14 | dCO | | SpringWheatNAM_tag_664 | | BS00049032_51 | 4.7 | 28.4 | -2.62 | cis | 3A | 17.079-23.905 |
|  | dCO | | SpringWheatNAM_tag_27582 | | Excalibur_c9348_504 | 3.1 | 16.2 | 1.98 | cis | 6B | 46.694-47.831 |
| NAM15 | pCO | | wsnp_Ku_c7811_13387117 | | Kukri_c2596_146 | 3.4 | 20.3 | 1.08 | trans | 3A | 120.843-152.794 |
| NAM16 | pCO | | wsnp_Ex_rep_c67100_65576619 | | D_GDRF1KQ02FFPXT_243 | 3.5 | 39.9 | 1.37 | cis | 6D | 46.666-116.526 |
|  | dCO | | Excalibur_c84439_196 | | SpringWheatNAM_tag_142344 | 3.1 | 18.8 | -2.06 | trans | 5A | 58.225 |
| NAM19 | pCO | | Ku_c70374_1383 | | IACX2158 | 3.1 | 24.1 | -1.46 | cis | 2D | 66.839-73.66 |
|  | dCO | | CAP8_c327_67 | | BobWhite_rep_c57286_122 | 3.2 | 17.8 | -1.84 | trans | 5A | 90.8215-93.664 |
| NAM20 | pCO | | BobWhite_c20282_164 | | BS00072157_51 | 3.5 | 21.2 | -1.18 | trans | 4A | 103.282-110.042 |
|  | dCO | | Excalibur_c8052_201 | | RFL_Contig5639_1168 | 3.3 | 21.0 | -1.95 | cis | 1D | 9.094-12.505 |
| NAM23 | pCO | | RAC875_c47084_378 | | BobWhite_c4004_61 | 3.2 | 16.1 | 1.04 | cis | 5B | 26.242-45.594 |
| NAM25 | dCO | | RAC875_c17347_216 | | RAC875_c17347_312 | 2.9 | 17.1 | 2.25 | cis | 6B | 29.634-80.808 |
| NAM28 | pCO | | wsnp_Ex_c1440_2764867 | | JD_c107_683 | 3.1 | 18.4 | 1.03 | cis | 1B | 48.9845-63.767 |
|  | dCO | | wsnp_Ex_c52577_56128947 | | Kukri_c77911_260 | 3.4 | 16.4 | -1.76 | trans | 6A | 47.3655-50.208 |
|  | dCO | | Ex_c31468_598 | | Ex_c6258_1094 | 3.2 | 15.5 | -1.71 | trans | 6D | 102.601-116.526 |
| NAM29 | pCO | | Excalibur_c8557_258 | | Kukri_c6014_816 | 4.3 | 17.6 | 1.07 | trans | 1B | 76.285-77.421 |
|  | pCO | | Excalibur_c26671_57 | | RAC875_rep_c109969_119 | 2.9 | 11.3 | -0.87 | trans | 5A | 47.99-51.401 |
|  | pCO | | Kukri_c24187_151 | | wsnp_Ra_c9155_15344108 | 3.2 | 12.9 | -0.93 | trans | 5B | 70.681-79.786 |
| NAM30 | pCO | | BobWhite_c26988_262 | | Kukri_c50943_853 | 3.7 | 16.0 | 1.01 | cis | 3B | 39.841-59.165 |
|  | pCO | | RAC875_c4792_754 | | RAC875_c43383_571 | 5.9 | 27.1 | -1.36 | cis | 5D | 159.327-161.6 |
|  | pCO | | Kukri_c438_1520 | | wsnp_Ex_c12781_20280445 | 4.1 | 31.1 | 1.43 | cis | 5D | 88.816-107.006 |
|  | dCO | | Kukri_c97631_275 | | wsnp_RFL_Contig3522_3685860 | 3.6 | 11.9 | -1.73 | trans | 2B | 59.752-72.825 |
|  | dCO | | Excalibur_c21964_237 | | wsnp_Ex_c41074_47987860 | 5.2 | 18.3 | -2.18 | trans | 4A | 91.787-96.361 |
|  | dCO | | BS00059503_51 | | RAC875_rep_c114716_194 | 10.4 | 43.4 | 3.28 | trans | 4A | 110.042-111.179 |

† W7984 location is the location of the highest associated marker within the detected QTL on the W7984 reference map

**Table S23**. Stepwise regression (SR) analysis results.

| Trait | Name | Chr | Position | Single Family Overlap (p<0.05) | Overlap p<0.0018 | df | SS | MS | F | pr>F | Model Rsq |
| --- | --- | --- | --- | --- | --- | --- | --- | --- | --- | --- | --- |
| TCO | SpringWheatNAM_tag_17106 | 1A | 49.06 | no | no | 28 | 2779.64 | 99.27 | 2.49 | 2.79E-05 | 0.4864 |
| TCO | SpringWheatNAM_tag_236732 | 1B | 44.44 | NAM15 | no | 28 | 2676.48 | 95.59 | 2.39 | 6.15E-05 | 0.4864 |
| TCO | SpringWheatNAM_tag_9254 | 2A | 59.23 | NAM1, NAM24 | NAM1 | 28 | 3799.40 | 135.69 | 3.40 | 6.10E-09 | 0.4864 |
| TCO | SpringWheatNAM_tag_246885 | 2A | 74.89 | NAM1, NAM24 | NAM1 | 28 | 2983.42 | 106.55 | 2.67 | 5.64E-06 | 0.4864 |
| TCO | SpringWheatNAM_tag_304176 | 2B | 72.83 | NAM17 | no | 28 | 2974.74 | 106.24 | 2.66 | 6.04E-06 | 0.4864 |
| TCO | SpringWheatNAM_tag_237047 | 4B | 50.38 | NAM19 | no | 28 | 2328.83 | 83.17 | 2.08 | 7.77E-04 | 0.4864 |
| TCO | SpringWheatNAM_tag_118459 | 5B | 38.77 | no | no | 28 | 2424.76 | 86.60 | 2.17 | 3.94E-04 | 0.4864 |
| TCO | SpringWheatNAM_tag_102677 | 5B | 38.2 | no | no | 28 | 2883.70 | 102.99 | 2.58 | 1.24E-05 | 0.4864 |
| TCO | SpringWheatNAM_tag_123291 | 5B | 69.54 | NAM18 | NAM18 | 28 | 2429.45 | 86.77 | 2.17 | 3.81E-04 | 0.4864 |
| TCO | SpringWheatNAM_tag_78137 | 5B | 50.14 | NAM18 | NAM18 | 28 | 3656.13 | 130.58 | 3.27 | 2.11E-08 | 0.4864 |
| TCO | SpringWheatNAM_tag_64311 | 5B | 134.5 | no | no | 28 | 3081.65 | 110.06 | 2.76 | 2.56E-06 | 0.4864 |
| TCO | SpringWheatNAM_tag_100953 | 5B | 144.8 | no | no | 28 | 3159.51 | 112.84 | 2.83 | 1.36E-06 | 0.4864 |
| TCO | SpringWheatNAM_tag_3087:55 | 6B | 47.83 | NAM4, 15 | no | 28 | 2629.87 | 93.92 | 2.35 | 8.73E-05 | 0.4864 |
| TCO | SpringWheatNAM_tag_80770 | 7B | 51.19 | NAM4,9,25 | NAM4 | 28 | 2857.17 | 102.04 | 2.56 | 1.53E-05 | 0.4864 |
| TCO | SpringWheatNAM_tag_102286 | 7D | 143.8 | no | no | 28 | 2493.60 | 89.06 | 2.23 | 2.39E-04 | 0.4864 |
| TCO | Error | -- | -- |  |  | 1652 | 65951.65 | 39.92 |  |  |  |
| pCO | SpringWheatNAM_tag_62074 | 1B | 75.15 | NAM29 | NA | 28 | 355.42 | 12.69 | 2.14 | 4.85E-04 | 0.3493 |
| pCO | SpringWheatNAM_tag_256152 | 2B | 61.46 | no | NA | 28 | 451.92 | 16.14 | 2.72 | 3.38E-06 | 0.3493 |
| pCO | SpringWheatNAM_tag_23856 | 2B | 72.83 | no | NA | 28 | 382.22 | 13.65 | 2.30 | 1.30E-04 | 0.3493 |
| pCO | SpringWheatNAM_tag_9157 | 3A | 20.49 | NAM11 | NA | 28 | 391.95 | 14.00 | 2.36 | 7.95E-05 | 0.3493 |
| pCO | SpringWheatNAM_tag_6621 | 3B | 62.58 | NAM30 | NA | 28 | 350.70 | 12.52 | 2.11 | 6.07E-04 | 0.3493 |
| pCO | SpringWheatNAM_tag_69842 | 6B | 44.42 | NAM5 | NA | 28 | 419.97 | 15.00 | 2.53 | 1.87E-05 | 0.3493 |
| pCO | SpringWheatNAM_tag_206798 | 6D | 110.6 | NAM16 | NA | 28 | 411.91 | 14.71 | 2.48 | 2.85E-05 | 0.3493 |
| pCO | SpringWheatNAM_tag_105073 | 7A | 4.551 | no | NA | 28 | 402.75 | 14.38 | 2.43 | 4.58E-05 | 0.3493 |
| pCO | SpringWheatNAM_tag_33172 | 7A | 63.95 | NAM13 | NA | 28 | 423.87 | 15.14 | 2.55 | 1.52E-05 | 0.3493 |
| pCO | SpringWheatNAM_tag_230837 | 7D | 148.4 | NAM8 | NA | 28 | 464.31 | 16.58 | 2.80 | 1.72E-06 | 0.3493 |
| pCO | Error | -- | -- |  |  | 1792 | 10621.76 | 5.93 |  |  |  |
| dCO | SpringWheatNAM_tag_155337 | 1B | 61.49 | no | NA | 28 | 1104.64 | 39.45 | 2.05 | 9.92E-04 | 0.3692 |
| dCO | SpringWheatNAM_tag_13579 | 1D | 21.61 | NAM20 | NA | 28 | 1282.74 | 45.81 | 2.38 | 6.73E-05 | 0.3692 |
| dCO | SpringWheatNAM_tag_236406 | 3A | 71.86 | no | NA | 28 | 1507.19 | 53.83 | 2.80 | 1.70E-06 | 0.3692 |
| dCO | SpringWheatNAM_tag_193560 | 3D | 67.32 | no | NA | 28 | 1131.89 | 40.42 | 2.10 | 6.67E-04 | 0.3692 |
| dCO | SpringWheatNAM_tag_189790 | 6B | 48.97 | NAM14 | NA | 28 | 1333.66 | 47.63 | 2.47 | 2.99E-05 | 0.3692 |
| dCO | SpringWheatNAM_tag_176391 | 7A | 81 | no | NA | 28 | 1106.21 | 39.51 | 2.05 | 9.70E-04 | 0.3692 |
| dCO | Error | -- | -- |  |  | 1904 | 36651.41 | 19.25 |  |  |  |

**Table S24.**  JCIM analyses results and effect size estimates for each family (separate Excel file, download from http://wheatgenomics.plantpath.ksu.edu/nam/).

**Table S25**. Marker estimates for significant Stepwise Regression markers by family (separate Excel file, download from http://wheatgenomics.plantpath.ksu.edu/nam/).

**Table S26**. Regions of interest detected in multiple scans and multiple components of recombination.

| **Chr** | **W7984 cM** | **Scans** | **RB Component** |
| --- | --- | --- | --- |
| 1A | 41.098-49.061 | TCO SR (49); Distal JICIM (41-44.5); Peri JICIM (44.5) | all 3 |
| 1B | 44.438 | TCO SR (44.4); Distal JICIM (44.4); Peri NAM5,13 | all 3 |
| 1B | 58.08-75.148 | Distal SR (61.5); Peri SR (75.1) NAM13,28 | pCO, dCO |
| 2A | 58.902-74.887 | TCO SR (59, 74), NAM1,24 | TCO |
| 2B | 59.184-72.825 | TCO JICIM (61), SR (72); Distal JICIM (59); Peri SR (61,72), JICIM (59) | all 3 |
| 3A | 17.079-20.49 | Distal NAM1,14; Peri SR (20), JICIM (18-20) | pCO, dCO |
| 3B | 54.619-62.576 | TCO JICIM (56-57); Peri SR (62) | TCO, pCO |
| 4A | 57.601-63.288 | Distal JICIM (57) | dCO |
| 4B | 50.376-58.338 | TCO SR (50); Distal JICIM (57-58) | TCO, dCO |
| 5B | 38.2005-69.5435 | TCO SR (38,50,69), JICIM (62); Peri NAM6,23,29 | TCO, pCO |
| 6B | 41.01-48.967 | TCO SR (47), NAM5,14,30; Distal SR (48), NAM8,14,25; Peri SR (44), NAM5,6 | all 3 |
| 7A | 0-4.551 | TCO NAM20,26; Distal JICIM (0); Peri SR (4.5) | all 3 |
| 7A | 63.946-84.41 | Distal SR (80.9); Peri SR (63) | pCO, dCO |
| 7B | 51.193 | TCO SR (51), JICIM (51), NAM4,9,25 | TCO |

**Table S27**. Conserved recombination candidate genes in regions of interest.

| **Chr** | **W7984 cM** | **Recombination Genes** | **Ortholog** |
| --- | --- | --- | --- |
| 1A | 44.512 | Traes_1AL_7242F453D | MutS |
| 1B | 44.438 | Traes_1BS_C6F54DA18 | RECQ Helicase |
| 1B | 58.08-75.148 |  |  |
| 2A | 59.228 | Traes_2AL_3446F3BC3, Traes_2AL_31566F0BB | MSH3, ZYP1 |
| 2B | 59.184 | Traes_2BL_1A30E0FF8, Traes_2BL_F49E62B6E, Traes_2BL_AD43FE11E | MSH3, ZYP1, PRD3 |
| 3A | 17.079-20.49 |  |  |
| 3B | 54.619-62.576 |  |  |
| 4A | 57.601 | Traes_4AS_5C5799ED6, Traes_4AL_5EE7ECC8A | SPO11-2, RECQ helicase l1 |
| 4B | 50.376 | Traes_4BS_D325D8F34, Traes_4BL_E74923982 (SPO11), Traes_4BS_8E7945BE5 (Rec) | FANCM, RAD54, TOP3A, RECQ helicase I1 |
| 5B | 39 | Traes_5BL_E1242566C | DMC1 |
| 6B | 47.831 | Traes_6BS_27266CEFE, Traes_6BS_3B5698FF2, Traes_6BL_F3A5A99E6 | HEI10, RAD23B-1, RAD54 |
| 7A | 0-4.551 |  |  |
| 7A | 82.137 | Traes_7AL_EC2DE4DFA | RAD51 |
| 7B | 51.193 | Traes_7BS_52A49E491 | RAD4 |

**Table S28**. Candidate genes with more than one homoeolog in a candidate region.

| **Wheat genes** | **Mapping approach** | **Map Location (Chr:cM)** | **Homologs** |
| --- | --- | --- | --- |
| Traes_1BS_85E907491 | NAM26, SR; | 1B:44.438 | PAX-interacting protein, PAXIP1 |
| Traes_1DS_344E5B51E | NAM2 | 1D:46.631 |  |
| Traes_2AL_31566F0BB | SR, NAM1, NAM24 | 2A:59.228 | ZYP1 |
| Traes_2BL_F49E62B6E | NAM17 | 2B:59.184 |  |
| Traes_2AS_42E368404 | SR, NAM1, NAM24 | 2A:59.228 | Abnormal spindle-like protein, ASPM |
| Traes_2BS_6374BF6AC | NAM17 | 2B:59.184 |  |
| Traes_2AL_3446F3BC3 | SR, NAM1, NAM24 | 2A:59.228 | DNA mismatch repair protein, MSH3 |
| Traes_2BL_1A30E0FF8 | NAM17 | 2B:59.184 |  |
| Traes_4AL_5EE7ECC8A | NAM16, Distal SR | 4A:57.601 | RecQ helicase |
| Traes_4BS_8E7945BE5 | SR, NAM19 | 4B:50.376 |  |
| Traes_5BL_E1242566C | SR, NAM18; | 5B:38-42 | DMC1 |
| Traes_5DL_F5BBB3008 | NAM26 | 5D:30.7 |  |
| Traes_6AS_E75394C0A | NAM4; | 6A:3.4 | CENP-O, kinetochore centromere component |
| Traes_6BS_1BC076E92 | NAM30 | 6B:2.3 |  |
| Traes_6AL_B13BFCFF8 | SR, NAM1, NAM24 | 6A:50.208 | HEI10 |
| Traes_6BS_27266CEFE | NAM5, NAM14, SR | 6B:47.831 |  |
| Traes_6AS_EE005D4DA | NAM18, NAM28; | 6A:48.0 | DNA repair protein RAD23b |
| Traes_6BS_89B173535 | NAM14, 24, SR; | 6B:47.8 |  |
| Traes_6BS_3B5698FF2 | NAM14, 24, SR; | 6B:47.8 |  |
| Traes_7AS_63EDF80FB | NAM26; | 7A:64.0 | DNA topoisomerase 1, TOP1 |
| Traes_7BS_3648891F6 | NAM25, NAM4, NAM9, SR, JCIM | 7B:51.2 |  |
| Traes_7AL_4DAAB4C8D | NAM26; | 7A:68.5 | Centromere / kinetochore protein zw10 |
| Traes_7BL_4A5D793C8 | NAM25 | 7B:52.3 |  |
| Traes_7BL_D90A3700B | NAM25, NAM4, NAM9, SR, JCIM | 7B:51.2 | DNA topoisomerase 1, TOP1 |
| Traes_7DL_EA65A81E5 | NAM25 | 7D:83.3 |  |
